# Protocol for a meta-analysis of stereotype threat in African Americans

**DOI:** 10.1371/journal.pone.0306030

**Published:** 2024-07-24

**Authors:** Russell T. Warne, Ross A. A. Larsen

**Affiliations:** 1 Independent Scholar, Columbus, Georgia, United States of America; 2 Department of Educational Leadership, Curriculum and Special Education, Arkansas State University, Jonesboro, Arkansas, United States of America; Northumbria University, UNITED KINGDOM

## Abstract

Stereotype threat is a well-known construct in psychology wherein individuals who belong to a negatively stereotyped demographic group underperform on cognitive or academic tasks due to the detrimental effects of a stereotype. Many psychologists have suggested that stereotype threat may be one of the reasons that some demographic groups are underrepresented in advanced academic programs and STEM fields. However, others have raised concerns about the quality of the stereotype threat research, suggesting that its apparent effects are inflated and that the phenomenon may be an illusion of questionable research practices and publication bias. The purpose of this proposed meta-analysis is to evaluate the existence of stereotype threat by (1) identifying the average effect size of stereotype threat studies in different types of studies, (2), investigating whether publication bias and *p*-hacking are present in the empirical research on stereotype threat, (3) testing for the influence of theoretical and methodological moderators, (4) assessing the overall quality of the research on stereotype threat, (5) and identifying the average effect in the methodologically strongest studies. This meta-analysis will be limited to studies that report data from African Americans because this population is a theoretically important group in stereotype threat research, and the size of score gaps between the African American and non-stereotyped populations in the United States should make the stereotype threat effect easiest to detect.

## Introduction

Stereotype threat is a phenomenon where members of a demographic group underperform on cognitive tasks when they are made aware of negative stereotypes about their demographic group. Ever since it was identified in a landmark article published by Steele and Aronson, [1] stereotype threat has been a widely accepted phenomenon in social psychology. Although the original research article on stereotype threat had African Americans as its target group, stereotype threat research has expanded to other groups, including females (e.g., [2,3]), Asian Americans (e.g., [4,5]), Hispanic Americans, [6] and others.

Steele and Aronson [1] matched African American and White participants on their SAT scores, exposed participants to a stereotype threat situation, and then observed a decrease in African Americans’ performance on academic tests. This design demonstrated that stereotype threat could create new score gaps between these two racial groups. [7] However, successful studies of interventions to counter stereotype threat [8] have provided evidence that has led many psychologists to believe that stereotype threat is a pervasive influence that is “in the air” [9, p. 613] and explains part of the average difference in academic performance between racial groups. [10,11] This makes stereotype threat a promising avenue for reducing average group differences on academic and cognitive tasks [8].

Steele [9] proposed three essential components that are necessary for stereotype threat to be activated in a person. First, the individual must identify with the subject domain of the cognitive task or test. This is what makes the stereotype into a threat; if a person cares little for a cognitive domain (e.g., math), then the idea that they will underperform because of their demographic group membership is not a concern. Second, the person must be aware of the stereotype, though it is not necessary for them to believe that it is true. This requirement also does not require the person to be consciously thinking about the stereotype during the cognitive task or test. Finally, the task must be sufficiently challenging that the person must expend cognitive effort—but not so difficult that the task is impossible for the person to accomplish. According to Steele’s [9] theory, without these three circumstances, stereotype threat will not be present and a person is much more likely to reach their optimal level of performance. Later researchers have built on this theory and often incorporate measurements of one or more of these characteristics into their investigations of stereotype threat [12].

Stereotype threat theory has important implications for society. [11] Most prominently, stereotype threat has been proposed as an explanation for part of the achievement gap between demographic groups on academic and intelligence tests [13] and one reason for the underrepresentation of some minority groups in academic pursuits, including advanced mathematics [14] and gifted programs [15]. Some psychologists have also claimed that the existence of stereotype threat is a serious problem that undermines the use and interpretation of cognitive and intelligence tests (e.g., [14,16]).

Stereotype threat has been extensively researched, and by 2012 (just 17 years after the original stereotype threat article was published), over 200 studies had shown the effect. [10, p. 147] In 2015, the tally had reached 300. [17, p. 2] Based on this large body of literature and resulting meta-analyses, stereotype threat is seen as a “robust” effect. [11, p. 418; 18, p. 7] The combination of a large body of research and the societal implications of the findings has made stereotype threat become a well-known topic in psychology. The phenomenon is a frequent topic of psychology education; three-quarters of introductory psychology textbooks cover stereotype threat. [19] Among rank-and-file psychologists, stereotype threat seems to have gained widespread acceptance.

### Stereotype threat theory and its discontents

While stereotype theory is widely endorsed, a growing cadre of researchers is questioning the implications, strength, and/or existence of the phenomenon. These critiques have arisen from empirical, methodological, and theoretical issues that have been difficult to square with the tenets of stereotype theory.

From an empirical perspective, much of the literature on stereotype threat is contradictory, [20] forcing the theory’s advocates to rely on interaction effects, mediators, and/or moderators to explain findings (e.g., [17,21]), even though these effects are less statistically stable than main effects. Moreover, given the supposed robustness of the phenomenon, a surprisingly large percentage of studies fail to show a statistically significant effect from stereotype threat. For example, in Shewach et al.’s [22] meta-analysis of stereotype threat effects, only 78 of 181 (43.1%) were statistically significant. Liu et al. similarly found only 49.2% tests were statistically significant. [8, p. 940] While these percentages are very weak evidence regarding the reality of the stereotype threat phenomenon, [23] they do show that approximately half of studies fail to detect the effect. In the face of frequent null findings, the meaning of many studies’ results is often unclear, and researchers should present the different scenarios that fit the data: a study with low statistical power, an effect or phenomenon that does not exist, potential confounding variables that made detecting an effect difficult, and more. Yet, when null findings occur, stereotype threat proponents often engage in *post hoc* rationalizations to salvage the theory and do not entertain the possibility that the phenomenon is not real (e.g., [4,24–27], though see [20,28] for authors who did consider that stereotype threat may not exist). These post hoc theories make stereotype threat adherents propose complicated boundary conditions to explain contradictions among studies (e.g., [11,12,17,18,29]). This criticism of stereotype threat research has been made before [30] and has never been fully addressed by stereotype threat advocates.

Methodologically, stereotype threat theory has not fared well in the wake of psychology’s replication crisis. In discussing research on stereotype threat research in females, Warne [31] found that the literature on the topic is dominated by research that has characteristics of studies that do not replicate, including small sample sizes, low statistical power, high researcher degrees of freedom, and the presence of publication bias (see also [20,32,33]). Large-scale, pre-registered studies of stereotype threat in adolescent female students have failed to detect the presence of stereotype threat in this population. [2,3] Most problematic for the theory is that studies that showed the presence of stereotype threat have not been successfully replicated in studies that closely follow the protocol of an earlier study [2,5,25] (though see [4] for a close replication that did partially support the earlier study’s findings). Even under a broad definition of “replication” that includes conceptual and theoretical replications (i.e., testing whether a study’s findings apply to a new population or a new setting, without closely adhering to the original study’s methodology), only 30–55% of replications could reproduce the findings of an earlier stereotype threat study. [34] For these reasons—and others—some scientists have suggested that stereotype threat evidence may have always been based on studies with weak methodology [20].

While advocates of stereotype threat theory may take issue with the claim that research on the topic has a poor replication record, a skeptical perspective is in harmony with the larger context of replications. In general, social psychology studies have a lower rate of successful replications than other fields, such as cognitive psychology [35] or personality psychology. [36] Additionally, stereotype threat experiments often use a form of social priming to trigger or ameliorate the phenomenon, [28] and other theories that rely on priming effects have had extremely poor records of replication (e.g., [37–38]).

There is also the possibility that methodological decisions in the original studies may inflate the frequency of false positives in the published literature (e.g., [34]). Indeed, evidence for *p*-hacking has been found in stereotype threat studies, [31,39] which would inflate the apparent strength of the evidence for stereotype threat. Support for this view is found in two recent meta-analyses of studies of stereotype threat, which both found that statistically significant results are much more likely in studies where the authors used statistical methods to adjust dependent variable scores. [28,34] This adjustment (especially in non-pre-registered studies) is an example of a questionable research practice that increases the likelihood of producing false positives. [40,41] One meta-analysis also indicates that other questionable research practices are common in the research on stereotype threat in females [34].

An additional characteristic that likely inflates the apparent strength of the stereotype threat phenomenon is publication bias. In examinations of previous studies pertaining to stereotype threat, numerous scholars have observed that the presence of publication bias within the academic literature is of sufficient concern. [22,24,28,31–33] Even modest levels of publication bias can greatly inflate the apparent average effect size of a body of studies. In simulation studies, moderate levels of publication bias were able to inflate an observed effect size in a meta-analysis from zero to *d* ≈ .30 or higher. [42,43] Given that stereotype threat proponents recognize that average effect sizes for the phenomenon are about *d* = .20 (e.g., [10,11,28]), it is legitimate to investigate whether, with current research characteristics, the strength of stereotype threat is overstated—or whether the phenomenon exists at all.

Another problem that is common in the stereotype threat research is that studies of the phenomenon often have characteristics (such as unusual test instructions or tasks) that were unrealistic in real academic settings. [28,44] In an analysis of this aspect of the research, Shewach et al. [22] found that studies with more ecologically valid characteristics were less likely to show a stereotype threat effect, a result that Priest et al. [28] found. Similarly, Liu et al. [8, p. 937] found that stereotype threat intervention studies in the field had effect sizes that were about half the size of lab-based studies. These findings support Lee’s suggestion that “… stereotype threat may be yet another curiosity of the psychological laboratory with minimal relevance to behavior in real-world situations.” [45, pp. 251, 252] A phenomenon that shows inconsistent results in the tightly-controlled environment of the laboratory and has an inflated evidence base is unlikely to have much impact in the real world.

A theoretical difficulty for stereotype threat theory is that it is not clear *how* activation of a stereotype leads to lower academic performance. [18] Some researchers have suggested that stereotype threat would consume working memory capacity, thereby reducing problem solving success (see also [13,46]). Another popular belief (e.g., [8,18]) is that the stereotype can trigger an anxiety response, which interferes with task performance. [18] The most informative study on how stereotype threat may function comes from a meta-analysis of interventions to counter stereotype threat. Each of the 11 intervention types were based on a different theoretical viewpoint of how stereotypes elicit lowered performance in study participants. The results of the meta-analysis showed that 9 of the 11 had statistically significant positive effects on stereotyped individuals. [8] A literature review of statistically significant mediator variables in stereotype threat research studies paints a similar picture: the researchers found 18 possible mediator variables for stereotype threat effects. [17] The multiplicity of possible mechanisms and mediators indicates that stereotype threat is, at best, a diffuse phenomenon and that there are many paths to a lowered score for stereotyped individuals (though under circumstances that can be difficult to predict). At worst, it is a sign of theoretical and empirical confusion, because it shows that scientists have little idea about how the phenomenon functions. To date, there is no consensus regarding the cognitive process(es) at work in a stereotyping situation that can result in lower academic performance and when a consistent stereotype threat effect can be triggered. Theorists’ strong reliance on mediators and/or moderators to explain when stereotype threat manifests itself (or not) undercuts the argument that stereotype threat is “robust.” As Rohrer et al. stated, “An effect cannot be both astonishingly robust and mysteriously fragile at the same time.” [38, p. 268]

### Purpose of this meta-analysis

Given the viable questions about the existence of stereotype threat, it is worthwhile to re-examine the evidence regarding the phenomenon. If stereotype threat is as robust and real as its proponents claim, then such an examination will dispel doubts about stereotype threat and permit examinations of effective interventions to combat its effects (e.g., [8]), or identify moderators [46] and mediators [17,18] for the effect. However, if stereotype threat is built on a foundation of poorly designed research, then such efforts are premature. Psychologists should prioritize conducting studies to enhance the evidential foundation concerning the phenomenon of stereotype threat before progressing to more intricate and advanced research topics associated with it.

Therefore, the purpose of this meta-analysis is to investigate the existence and strength of stereotype threat in African Americans. In particular, the goals will be to (1) establish the average observed effect size and heterogeneity of effect sizes in different types of stereotype threat studies, (2) investigate whether publication bias and/or *p*-hacking are present in this literature, (3) examine whether study characteristic moderator variables explain heterogeneity among effect sizes, (4) ascertain the overall quality of the research on stereotype threat in African Americans, and (5) estimate the average effect in the best designed studies. By making this meta-analysis a registered report, researcher subjectivity will be minimized, and the results will be more trustworthy for everyone, regardless of their preconceived beliefs about stereotype threat.

This meta-analysis will focus on African Americans for four reasons. First, in comparison to other major racial and ethnic groups in the United States, African Americans exhibit the most pronounced score differentials on cognitive and academic assessments, alongside the lowest levels of representation in higher education and advanced academic programs. [47–49] Therefore, if stereotype threat is a real phenomenon that depresses academic performance, it should be easiest to detect in this demographic group. Conversely, combining African Americans’ data with other demographic groups’ data may dilute the effect sizes and make stereotype threat harder to detect, especially if—as has been suggested—stereotype threat mechanisms are heterogeneous across populations [17].

Second, in the initial identification of stereotype threat, its manifestation was observed in African American examinees. [1] Consequently, it is imperative to meticulously ascertain the presence and intensity of stereotype threat within this demographic before generalizing its applicability to other groups. Furthermore, recent studies have cast doubt on the prevalence of stereotype threat in females [2,3,18,31], raising the legitimate prospect that this phenomenon may not manifest in various populations, including African Americans.

Third, only two prior meta-analyses on stereotype threat in racial or ethnic groups (by Nadler and Clark [6] and Priest et al. [28]) have reported results separately for African Americans. However, in both of these meta-analyses, there were a the limited number of studies, comprising 16 for African Americans and 13 for Hispanics in Nadler and Clark’s [6] meta-analysis and just 10 studies in the Priest et al. [28] meta-analysis. This may have constrained the statistical power of this analysis. The reports of both of these meta-analyses make it likely that there are additional studies that could be included in a meta-analysis on stereotype threat in African Americans. Nadler and Clark’s work was published 13 years ago, and Priest et al.’s search procedure [28, p. 6] consisted of a few Google Scholar searches and contacting study authors for unpublished work. We believe that a more thorough search will yield more studies eligible for inclusion and a larger evidence base regarding stereotype threat in African Americans.

In the years since Nalder and Clark’s article, meta-analysis practices—often in response to the replication crisis in psychology—have advanced considerably. Nadler and Clark’s [6] meta-analysis was adequate for the standards of its time, but there was no attempt to identify publication bias or questionable research practices that could inflate the average effect size of a meta-analysis. Other authors of meta-analyses have identified the possibility of publication bias in the stereotype threat literature, [22,28,31–33] and it would be important to know how severe of a problem this is in the studies of African American participants.

Finally, some scholars argue that the African American experience diverges from that of other racial and ethnic minorities in the United States, potentially giving rise to distinctive factors that diminish and/or underestimate their cognitive accomplishments (e.g., [50,51]). For instance, Ogbu [51] contends that African Americans are categorized as an "involuntary minority," frequently encountering exclusion and marginalization, coupled with the devaluation of their cultural heritage. This stands in contrast to "voluntary minorities" who choose to immigrate and embrace the values of a host country, often leading to a more rapid assimilation and acceptance by the majority culture. If this assertion holds true, there is both theoretical and practical merit in isolating the examination of stereotype threat within the African American context. The impact of stereotype threat on this population is posited to differ in terms of causation and/or intensity compared to other groups facing stereotype threat (e.g., Hispanic Americans, females). Advocates for the stereotype threat concept suggest that demographic groups exhibit heterogeneity in the types of threats to which they are vulnerable [17]. Consequently, a meta-analysis exclusively focusing on African Americans should enhance the understanding of stereotype threat dynamics.

### Hypotheses

Our principal hypothesis for this meta-analysis is that there will be an overall non-zero average effect (two-tailed *p* < .05), showing evidence for the existence of stereotype threat in African Americans and that a statistically significant level of heterogeneity will exist among effect sizes. This belief is based on prior work showing a stereotype threat in this demographic group (most notably, [6]) and for the fact that publication bias and questionable research practices can greatly inflate effects and lead to a surplus of false positives. Our second hypothesis is that there will be sufficient evidence for publication bias and/or questionable research practices (e.g., *p*-hacking) and that these methodological artifacts would be strong enough that they could produce the apparent overall average effect size in the meta-analysis. Our third hypothesis is that there will not be sufficient evidence for any moderator variables to explain a non-trivial amount (*R*^2^ change ≥ .10; as we are using multiple regression) of variance in effect sizes. Our fourth hypothesis is that the quality of the research on stereotype threat in African Americans will be generally poor. Finally, we hypothesize that the evidence in favor of the phenomenon weakens as research quality increases to the point that the effect size based on the best studies is not statistically significant (with α = .05, using a two-tailed test of statistical significance).

## Methods

This meta-analysis has been planned to conform to the standards of the Preferred Reporting Items for Systematic reviews and Meta-Analyses for Protocols (PRISMA-P; see [52]), with some additional information based on the Generalized Systematic Review Registration Form template. [53] The final results are intended to be reported in conformity to the PRISMA guidelines.

### Search procedure

The process to identify published studies on the stereotype threat phenomenon in multiple stages. In the first stage, there will be a search of APA PsycArticles, APA PsycInfo, JSTOR, SAGE Journals, Scopus, and Web of Science databases for the phrase “stereotype threat” and either the word Africa* or Black* in the abstract. Note that JSTOR has poor coverage of article abstracts. Therefore, in this database, the search will be for the phrase “stereotype threat” in titles.

The second phase will be a search for the phrase “stereotype threat” in unpublished studies by using the Open Science Foundation’s search tool (https://osf.io/preprints/discover) in nearly 2.4 million research documents in 34 pre-print repositories, including PsyArXiv and EdArXiv. There will also be a search of the ProQuest dissertation and thesis database for the phrase “stereotype threat” and either the word Africa* or Black* in the abstract. Finally, there will be a search Google Scholar for the same phrase and keywords and examine the first 1,000 results to identify unpublished studies and studies that did not appear in any earlier searches.

In the third phase of the search, we will contact researchers to identify additional studies and data sources that can be included in the meta-analysis. This will involve contacting authors of prior meta-analyses to ask for any unpublished studies they have. We will also contact researchers who have published stereotype threat studies in African Americans and request the data or summary statistics from any unpublished studies. This request will also extend to studies that did not meet inclusion criteria as published but would meet inclusion criteria if the raw data were made available to us. For example, if a published article only reports results with data from Hispanics and African American participants combined into a single sample, (e.g., [54]), we will ask the corresponding author for the raw data so that we can include data from just African Americans in our meta-analysis.

In the final phase of the search, studies that reported data from racial minorities in earlier meta-analyses will be examined to determine whether they reported data for African Americans. Likewise, the reference list of identified articles will also be examined to search for additional studies that report stereotype threat research in African Americans, and a cited reference search of the original Steele and Aronson article [1] and Steele’s theoretical paper [9] will be used to identify additional studies of stereotype threat in African Americans.

Information on publications and reports will be recorded in a shared Google spreadsheet with automatic version control. Publications and reports will indicate how a record for a study was obtained and whether it met the inclusion and exclusion criteria. The primary reason for exclusion from the study will be recorded for studies that do not meet the inclusion and exclusion criteria. The shared spreadsheet will also report the results of our attempts to contact authors of studies and meta-analyses of stereotype threat in African Americans.

### Screening studies

All studies will be screened by a qualified individual with advanced training in psychology research methodology (a PhD-holder with specialized training in quantitative methodology) for inclusion. Records will be screened first by title and abstract and then by reading the full text of the report. Uncertainty about whether a study or publication meets the inclusion and exclusion criteria will be resolved through team discussion.

If a study has been published more than once (e.g., as a dissertation and as a journal article, or a longitudinal study that reports outcomes separately at the end of an intervention and also at a later time point), the more complete version of the study will be included. For longitudinal studies, this will be the final available report. For one-time or cross-sectional studies, this will be the version that reports the largest sample size. If a record appears in more than one database search, then we will identify duplicates manually by matching DOIs; for records that do not have a DOI attached, then matching author names and record title (and other study characteristics, such as sample size or identical methodology) will be used to identify duplicates. All records will be recorded in a shared Google spreadsheet with automatic version control.

All screening will be conducted by one of two senior scientists with a PhD in psychology and advanced training in quantitative methodology. Both screeners are co-authors on this registered report protocol and are, therefore, not blinded to the study’s hypotheses. To assess screening accuracy, a random 10% of records will be checked by the other screener. We will calculate their percentage of agreement to measure the reliability of the screening process. Disagreements will be addressed via discussion. If screening agreement drops below 90%, we will reassess why agreement is too low and refine the screening process.

### Inclusion and exclusion criteria

After studies have been collected, they will be screened for inclusion in the meta-analysis. There are nine inclusion/exclusion criteria explained in this section. Any study that does not have all of these characteristics will be excluded from the meta-analysis.

#### Participants

The study must report data for African Americans, and these data cannot be aggregated with any other racial, ethnic, or cultural group.

#### Stereotype threat intervention

The study reports a stereotype threat intervention that is intended to either (a) trigger a decrease in examinees’ performance, or (b) counteract or mitigate the effect of stereotype threat that is presumed to be pre-existing.

#### Dependent variable

The study must have a post-test dependent variable that is an academic or cognitive measure of aptitude or achievement, such as a test score, grades, or academic ratings from an observer or teacher.

#### Effect size

To be included in the meta-analysis, a study must report an unadjusted effect size or sufficient summary data to calculate an effect size. Adjustments that will disqualify effect sizes are ANCOVA, *pre-test score x group* interactions, and any other statistical adjustment that is applied to post-test data before calculating effect sizes (see [20] for a similar exclusion criterion).

#### Research design

Another inclusion criterion is that the research design for the study must have a comparison group. These research designs can take three forms that each produce a statistical main effect:

1. A within-subjects design where pre-test scores function as a control group for the same African American participants who are later exposed to the intervention,
2. A between-subjects design where a control group of African American participants are a comparison group for African Americans exposed to a stereotype threat intervention, or
3. A between-subjects design comparing outcomes for African Americans exposed to stereotype threat intervention and a group of participants who are not the target of negative stereotypes but who are also exposed to the stereotype threat intervention; in most studies these will mostly be White participants.

Note that the choice of comparison group impacts the conclusions that can be drawn about stereotype threat. The first two designs can provide evidence that a stereotype threat intervention impacts African Americans’ scores, whereas conclusions about whether stereotype threat contributes to interracial differences in a dependent variable are only possible with a comparison group of participants who are not the target of a negative stereotype (i.e., the third design).

All three of the above comparison groups can produce a statistical main effect that provides insight into the stereotype threat phenomenon. However, many studies (including the original stereotype threat studies from Steele and Aronson [1]) examined interaction effects that can also provide insight into stereotype threat. Continuing the above numbering, these interactions can be any of the following:

4. A *time x group* interaction effect that arises when the experimental and control groups of African Americans take a pre-test and post-test, with the experimental group receiving a stereotype threat intervention between the tests;
5. A *race x group* interaction effect when two racial groups (African Americans and non-stereotyped participants) belong to an experimental group that receives a stereotype threat intervention, with a control group consisting of individuals from both racial groups serving as a comparison group; or
6. A *race x time x group* interaction that can occur when a study design includes a pre-test and a non-stereotyped comparison group.

Again, it is important for readers to note that these interaction effects produce different conclusions about stereotype threat. The *time x group* interaction permits conclusions about the magnitude of intra-individual change that occurs as a result of the stereotype threat trigger or intervention, compared to a baseline group’s change over the same time period. This allows a researcher to eliminate maturation and practice effects as possible threats of validity, compared to the within-persons design that lacks a control group. (This design does not eliminate the possibility of expectancy effects in the experimental group, though.) The *race x group* interaction permits conclusions about the differential impact of the stereotype threat intervention on different racial groups, which eliminates the possibility that pre-existing racial group differences cause later differences in the dependent variable. (Random assignment of participants within a racial group to the experimental and control groups is an additional requirement for eliminating this threat to validity.) Finally, the three-way interaction design (*race x time x group*) would permit conclusions that interracial differences in the dependent variable change from pre-test to post-test when African Americans receive a stereotype threat intervention. [41, p. 542].

All these designs will be permitted in the meta-analysis. However, their effect sizes will be coded and meta-analyzed separately because the designs do not produce results that are interchangeable. For example, a study that produces a *race x group* interaction will have that effect size coded separately from its main effect estimated through the intercept *group*. The two different effect sizes will also be meta-analyzed separately and will not be averaged together.

#### Random assignment

With the exception of the within-persons design (see the initial design in the list), all African American participants must be randomly assigned to at least one group that receives an experimental treatment. Correlational designs and studies that do not randomly assign African Americans at the individual or group-level (e.g., by applying random assignment to an entire classroom or testing session) to a treatment or control group will be excluded from the meta-analysis. This will ensure that the intervention to trigger or mitigate stereotype threat has a causal impact on the dependent variable(s) and will distinguish this meta-analysis from recent ones on stereotype threat that have included correlational designs [22] and quasi-experimental designs [8].

#### Year of study

A study must be published or made available (e.g., as a pre-print or a dissertation) between 1995 (when the original stereotype threat study was published) and 2023. Unpublished studies that are not pre-prints or dissertations must be conducted between 1995 and 2023.

#### Location of study

Because the focus of this meta-analysis is African Americans, the study must be conducted in the United States.

#### Language of report

The report for a study must be written in English.

#### Coding

Following identification, the studies will undergo a systematic coding process to generate data for subsequent meta-analysis. The coded variables are distributed across eleven distinct categories, namely: (1) document/study information; (2) setting; (3) independent variable details; (4) information pertaining to intervening, moderating, and mediating variables; (5) details on dependent variables; (6) characteristics of the sampled population; (7) reported results; (8) statistical power; (9) data/sample member exclusions; (10) covariate control; and (11) miscellaneous notes.

The selection of these coding variables was guided by their necessity for facilitating a comprehensive meta-analysis: theoretical significance (e.g., adherence to Steele’s [9] prerequisites for the occurrence of stereotype threat, all of which are coded as moderator variables), relevance in previous meta-analyses (e.g., the incorporation of a statistical method for controlling group differences; refer to [28,34]), or statistical importance (e.g., inclusion of statistical power information). A comprehensive list of variables and their respective coding guidelines can be found in S1 Appendix.

#### Data accuracy

To maximize data accuracy, all studies that meet the inclusion criteria will be coded by one of two team members with advanced methodological training in quantitative methods in psychology. When this coder has finished, a second coder with the same credentials will independently code each study using the same text and/or data. Discrepancies will be resolved through discussion. The percentage of initial agreement between the two coders will be calculated for every variable and reported in the final document. Both data coders are co-authors on this registered report protocol and are, therefore, not blinded to the study’s hypotheses.

#### Data dependence

It is likely that there will be dependence in the meta-analytic data due to (1) the same document reporting multiple studies, and (2) the same study reporting multiple effect sizes. Data dependence can be managed at two stages of a meta-analysis: coding and analysis. During the coding process, we will record data at the most granular level reported that still includes all sample members. For example, Hollis-Sawyer and Sawyer [21] reported data separately for participants who were exposed to a high face validity condition and participants who were given a low face validity condition. In this case, we will record data for the two independent samples—one for each condition. This procedure will permit us to report the most granular data possible within studies so that the fullest possible heterogeneity of effect sizes is available to our analyses.

A special case of subgroup analysis arises when considering reports from racial groups; it is possible that some researchers may report data separately for different racial groups. [e.g., 21] Because African Americans are the focal group in this meta-analysis, any race main effects or interaction effects where race is a main variable will be reported separately for comparing African Americans to non-stereotyped groups. Any other racial comparisons will not be included in our dataset. For example, if a researcher reports means and standard deviations for African Americans, Hispanics, Whites, and Asians, then we will record the effect sizes that show the dependent variable difference between African Americans and Whites and between African Americans and Asians. No data from Hispanics will be recorded (as they do not belong to a non-stereotyped group, nor are they the focal group of interest in our meta-analysis), and no comparisons between Whites and Asians will be recorded (because this comparison does not include African Americans).

During the data analysis process, we will deal with this dependence in the data using a multilevel random effects model will be used to apportion variance into three levels: the sampling variance for each effect size within the same study (level 1), the variance among studies (level 2), and variance across articles that contain several studies (level 3). We will employ a multilevel random effects model regardless of the values of the heterogeneity statistics. The multilevel random effects model is chosen for its ability to yield robust results in both high and low heterogeneity scenarios.

#### Missing data

Because a study design can have up to 67 variables coded (see S1 Appendix), it is likely that there will be missing data. When a research report indicates that a missing variable may have been collected but unreported (or insufficiently reported), we will contact the author(s) to request the needed data. When the missing data seems unlikely to have been collected or an author is unavailable or nonresponsive, we will use the full-information maximum likelihood technique to keep all subjects in the analysis [55].

#### Statistical analysis

Effect size coding. Main effects derived from Research Designs 1–3 will be recorded as Cohen’s *d* values. For the within-group design, the denominator for the effect size will be the standard deviation of the sample’s pre-test scores. For the between-group designs, the denominator will be the pooled standard deviation of the two groups. Effect sizes for the interactions will be recorded as η^2^ values. To facilitate comparisons of effect sizes across main effects and interactions, the Cohen’s *d* values for the main effects will also be converted to η^2^ values.

#### Mean effect size and effect size heterogeneity

The statistical analysis will be conducted in the metafor package in R by one co-author, with the other co-author checking the analysis for accuracy and reproducibility later. [56] An overall average effect size and heterogeneity statistics will be calculated for studies that meet the inclusion criteria, with one average effect size (and accompanying heterogeneity statistics) calculated for each of the six designs. The results will be summarized as six effect sizes because each of the six designs listed above provides evidence about different aspects about stereotype threat theory and the designs, their results, and the conclusions that one can draw from them are not interchangeable (see [28], for an example of a meta-analysis that reports different average effect sizes for different study designs).

#### Heterogeneity statistics

Three commonly used heterogeneity statistics will be reported and interpreted (*Q*, *I*^2^, τ^2^). If these statistics disagree, we will defer to τ^2^, as it is the most robust to both within-study sample size and number of studies in a meta-analysis. [57].

#### Moderator analysis

After the mean effect sizes are calculated for each design, each will be subjected to a moderator analysis to determine whether study characteristics influence the magnitude of the effect size. A total of 28 variables that could serve as study characteristic moderators in the meta-analysis are marked in the coding document (see S1 Appendix). These study characteristic moderators selected for this purpose are listed in S2 Appendix, along with the reasons for their selection and supporting references (where applicable) for why they are plausible moderator variables. These variables fall into four categories: (1) theoretically relevant moderator variables, (2) methodologically relevant variables, (3) empirically relevant moderator variables, and (4), exploratory moderator variables.

The theoretically relevant variables are potential moderators because stereotype threat theorists have posited that these variables can impact the presence and/or magnitude of the stereotype effect phenomenon. For example, because Steele [9] theorized that stereotype threat occurs when there is (1) an awareness of the stereotype, (2) self-identification from the examinee towards the domain of the cognitive task, and (3) a task that is of moderate difficulty, these are theoretical moderators that could influence the magnitude of a study’s effect size. Indeed, Picho-Kiroga et al. [12] used these study characteristics in a moderator in their meta-analysis. Methodologically relevant variables are those which have been shown in research on publication bias or the replication crisis in psychology as having an influence on effect sizes in studies. Empirically relevant variables are those that have been shown to be important moderators in prior meta-analyses that have examined publication bias with modern methods. Finally, we have three exploratory variables that we are including because of our methodological interests (see S2 Appendix).

Like any statistical procedure, moderator analysis is most informative with a large dataset. Therefore, it is prudent to set minimum sample size requirements *a priori* in order to increase the likelihood that the analyses will be informative. In our meta-analysis, a moderator analysis will only be conducted for study characteristic moderator variables that have been reported and coded for at least 15 studies or 25% of studies (whichever value is larger) and there are at least two values for the moderator variable that each are recorded for at least 6 studies or 10% of eligible studies (whichever value is larger) within a study type. Moderator analyses for variables that do not meet these arbitrary thresholds will not be performed. Readers should notice that these values are at the study level. Consequently, if the same study provides multiple effect sizes for the meta-analysis, then it will only count once towards meeting the thresholds listed above.

Moderator variables that meet this threshold will be tested individually to determine whether the study characteristic is associated with a systematic difference in effect sizes across studies. The tests will be non-directional with an α value of .05, reflecting the fact that we have no strong hypotheses about the results of the moderator analyses. The standardized betas will also be calculated and graphs that show the nature of the statistically significant interaction(s) (if any) will be produced. Moreover, we will employ the *Q*-test procedure, which assesses the moderator effect through subgroup analysis and the chi-square distribution. This will serve to corroborate the traditional *t*-test associated with the beta of the interaction [58].

Testing a large number of moderators individually can be statistically problematic for two reasons. First, testing many moderators individually will increase the risk of a Type I error. Second, when moderators are highly correlated, the individual tests for their statistical significance may indicate an independent effect that is illusionary. Correlated moderators can occur when the number of studies is small, or when study characteristics co-occur frequently because authors make similar methodological decisions (e.g., if the random assignment and intervention both occur at the group level). To be aware of this possibility, we will report a Cramer’s *V* value for every pair of variables that are the target of a moderator analysis. An alternative option would be to perform a meta-regression, but we doubt that this meta-analysis will report a sufficient number of studies to make that option feasible (see [59] for the sample size needs of meta-regression).

#### Statistical model

The choice of the data analysis model hinges on various factors, including the research design and the presence of potential moderators. In the simplest case—in which the study lacks measurements for potential moderators, employs a non-longitudinal design, and exclusively involves African-Americans—a straightforward three-level multilevel regression (where effect sizes are clustered within studies, which are clustered within publications) with a simple linear structure will be applied. This model includes a random effect for variation within studies and variation between studies, although it is not explicitly presented here for the sake of brevity (see S3 Appendix for a more detailed treatment).


$$y_{i}=\beta_{0}+\beta_{1}{\left(Intervention\right)}_{i}+\varepsilon_{i}$$

In this academic study, we denote the observed effect size for individual study *i* as *y*_*i*_. The term *β*_*0*_ represents the average test scores from the control group, while *β*_*1*_ signifies the treatment effect of an intervention aimed at mitigating or triggering stereotype threat. The residual for individual study *i*, denoted as *ε*_*i*_, is derived from the difference between the expected and observed test scores for that individual study. We will rigorously assess the assumptions underpinning our model by employing a diverse array of diagnostic tools. These encompass residual plots to evaluate linearity, autocorrelation plots to scrutinize independence, and histograms of residuals to assess normality and detect outliers. Furthermore, we will utilize model fit statistics, including (*R*^2^), and employ variance inflation factor (VIF) statistics to gauge multicollinearity. The Durbin-Watson test will be applied to detect autocorrelation, while Q-Q plots will aid in verifying the normality of residuals. Additionally, to ensure temporal stability, we will conduct subgroup analyses based on study publication year.

To assess stereotype threat, *β*_*0*_ serves as a key parameter. It enables the calculation of the average test scores for an untreated group of African Americans, allowing for a meaningful comparison with a comparison group of African Americans who have not been exposed to the stereotype threat intervention.

In more complex scenarios, where the study encompasses multiple ethnic groups, the multilevel model can be expanded as follows:

$$y_{i}=\beta_{0}+\beta_{1}{\left(Intervention\right)}_{i}+\beta_{2}{\left(African-American\right)}_{i}+\beta_{3}{\left(African-American*Intervention\right)}_{i}+\varepsilon_{i}$$

Where *β*_*2*_ specifically captures the estimate for African American group membership and serves as an estimate of the stereotype threat effect. The parameter estimate *β*_*3*_ calculates the differential effect of the treatment on African Americans. These examples illustrate the models employed to discern and quantify stereotype threat, with the number of control terms varying based on the research design and the presence of other pertinent variables, as previously mentioned.

#### Statistical power analysis

Previous researchers examining the stereotype threat literature have criticized the low statistical power often found in stereotype threat studies (e.g., [22,31,32,44]), and even some proponents of stereotype threat theory have recognized this shortcoming in the research. [12,14] To examine this issue, any calculations of *a priori* statistical power that study authors have made will be recorded, along with calculations of statistical power for each study calculated specifically for this meta-analysis. The *a priori* calculations for this meta-analysis will be based on a main effect size of *d* = .20, which is typical mean effect size for stereotype threat meta-analyses and literature reviews (e.g., [11,14,28,29,60]). For calculating the statistical power of interaction effects, the effect size used will be η^2^ = .001 (mathematically equivalent to *d* = .20; see [61, p. 303]), with the assumption of an accompanying null main effect. All statistical power calculations will be based on two-tailed tests with α = .05. After statistical power values have been recorded and/or calculated, the mean power for each group of effect sizes (i.e., the mean effect size for each of the six designs) will be reported.

#### Tests of publication bias and examinations of *p*-hacking

In this meta-analysis, there will be five reported tests of publication bias: (1) a funnel plot, accompanied with Egger’s test of publication bias; (2) Ioannidis and Trikalinos’s [62] test of excessive significance; (3) Simonsohn et al.’s [41,63] *p*-curve; (4) Bartoš and Schimmack’s [64,65] *z*-curve (implemented through the zcurve package in R); and (5) PET-PEESE. If there are at least 15 studies with the same type of design, then the tests will be conducted on the subset of effect sizes for each design type, though these will be interpreted tentatively because the smaller number of effect sizes will give these tests less statistical power to detect publication bias. It will be concluded that publication bias is likely enough to be concerning if at least three of these techniques show statistically significant evidence of publication bias in a set of effect sizes. This standard is needed because none of these methods is—by itself—can provide conclusive evidence of publication bias. [66] Note that an assumption of the *p*-curve is that contributing *p*-values are statistically independent of one another. When researchers investigate interaction effects, but the meta-analyst is interested in main effects, this assumption is violated [40, pp. 543–544; 63, p. 675]. This will be taken into account when selecting *p*-values for the *p*-curve analysis.

None of these methods of detecting publication bias functions well when effect sizes are clustered within studies and between-study heterogeneity (and publications) is high. [67] If this is observed in our multilevel random-effects model (as defined by an *I*^2^ value of 65% or above), then it will be necessary to create a new multilevel meta-regression model to test for publication bias. In this model, we will use the standard errors of the effects at the study level as predictors, [67] which will produce a more accurate estimate for the magnitude of effect size inflation due to publication bias. Readers should also note that some of these tests of publication bias also can provide circumstantial evidence for *p*-hacking. Indeed, often it is not possible in a meta-analysis to determine whether a surplus of statistically significant results is the result of publication bias, *p*-hacking, or both. This is because authors’ and journal editors’ belief that a statistically significant result is more publishable (which causes publication bias) incentivizes *p*-hacking. Neither publication bias nor *p*-hacking occurs in a vacuum.

Researchers familiar with prior meta-analyses of the stereotype bias literature will notice that the three most common methods of testing for publication bias in the field are missing from this pre-registration: the fail-safe *N* procedure, Begg’s test of publication bias, and the trim-and-fill method. These are omitted because they are uninformative, due to their unrealistic statistical assumptions. Fail-safe *N* estimates the number of null studies omitted from the literature in order for the observed mean effect size to be found if the true effect size is zero. However, when publication bias and *p*-hacking occur, it is not the entire study that is omitted, but rather the results of a particular analysis. [41] Thus, the fail-safe *N* value is not the number of missing studies, but the number of missing *analyses*. Because the number of unreported and “unsuccessful” analyses (i.e., analyses that produce a statistically insignificant result) in the stereotype threat literature cannot be known, it is impossible to judge whether the results of the fail-safe *N* procedure indicate a plausible number of omitted analyses for publication bias to explain the observed effects of a meta-analysis. Begg’s test is not included because it has extremely low statistical power for detecting typical levels of publication bias [68] and is therefore rarely informative. Finally, the trim-and-fill method will not be performed because its underlying assumption is that publication bias arises because studies remain unpublished because of a small effect size. However, in reality, it is studies’ *p*-value—not their effect size—that is the most powerful influence on publication bias. Moreover, the trim-and-fill method severely undercorrects in simulations with known publication bias in a body of effect sizes. [63]

### Judging overall quality of stereotype threat research

To ascertain the overall quality of research on stereotype threat in African Americans, we will calculate a methodological quality score for each study based on data extracted from the coding process. The scoring system is shown in Table 1 and is based solely on methodological considerations—thereby increasing objectivity. In this system, studies can obtain a maximum of 16 points. Following the example of [69], we will conduct a subgroup analysis of studies of increasing standards of quality. We have labeled these standards as:

- basic standards of quality (defined as a score of at least 4 points),
- moderate standards of quality (defined as a score of at least 8 points), and
- high standards of quality (defined as a score of at least 12 points).

**Table 1:** Scoring System for Methodological Quality of Studies.

Criterion	Scoring of Criterion
Pre-registration	Pre-registration dated before data collection and is followed closely: 1 point
Replication	Study was a close replication of a prior stereotype threat study: 1 point
Control group	Active control group is part of study: 1 point
Power analysis	*A priori* power analysis conducted before data collection: 1 point
Statistical power^a^	≥ .80: 2 points ≥ .50: 1 point
Dependent variable scoring	Scoring is blind or objective: 1 point
Setting	Realistic or ecologically valid setting (i.e., school/classroom, clinic, or other location where participant would expect to be evaluated) for experiment: 1 point
Essential conditions^b^	Majority of African American subjects are aware of stereotype: 1 point Majority of African American subjects identify with the domain tested in the experiment: 1 point Researcher(s) showed that the task is moderately difficult (i.e., no floor or ceiling effects) for all groups of African Americans: 1 point
Manipulation check	Manipulation check is present: 1 point
Type of dependent variable	Dependent variable is an unaltered standardized test score: 1 point
Stakes of dependent variable	Dependent variable has high-stakes consequences for a majority of research participants: 1 point
Open materials	Study materials (e.g., stimuli, experimental procedures) are freely available for download: 1 point
Open data	All study data are freely available for download: 1 point

*Note*. Maximum score is 16 points.

^a^ Maximum criterion score is 2 points; effect size calculated based on *d* = .20.

^b^ Maximum criterion score is 3 points.

This step-up procedure will allow us to draw inferences about the apparent magnitude of effect sizes and whether this decreases as methodological quality of a study increases. This subgroup analysis will also permit a conclusion about the quality of the total body of research on stereotype threat in African Americans and an overall assessment of the evidence for the existence of stereotype threat based on the strongest studies.

### Adjustments to the protocol

If a desire for unforeseen adjustments to the protocol arises, all work on the meta-analysis will stop, and the academic editor assigned by *PLoS ONE* to the study will be contacted. The proposed change(s) to the protocol will be explained to the academic editor, who will make a decision regarding whether the proposed change(s) is/are acceptable. Work will not resume until the academic editor has rendered a decision. Any progress on the meta-analysis that occurs after the decision will conform to the academic editor’s decision. All adjustments will be noted in the final text of the study report.

### Open science practices

We have designed this meta-analysis to conform to all open science practices. This published Registered Report protocol will function as the study’s pre-registration. All data and statistical analysis code will be made publicly available upon final publication at the Open Science Foundation’s repository at https://osf.io/.

## Supporting information

S1 Checklist PRISMA-P 2015 checklist. (DOCX)

S1 Appendix (DOCX)

S2 Appendix (DOCX)

S3 Appendix (DOCX)

## References

[1] Steele CM, Aronson J. Stereotype threat and the intellectual test performance of African Americans. J Pers Soc Psychol. 1995;69: 797–811. doi: 10.1037//0022-3514.69.5.797 7473032

[2] Finnigan KM, Corker KS. Do performance avoidance goals moderate the effect of different types of stereotype threat on women’s math performance? J Res Pers. 2016;63: 36–43. doi: 10.1016/j.jrp.2016.05.009

[3] Flore PC, Mulder J, Wicherts JM. The influence of gender stereotype threat on mathematics test scores of Dutch high school students: A registered report. Compr Results Soc Psychol. 2018;3: 140–174. doi: 10.1080/23743603.2018.1559647

[4] Gibson CE, Losee J, Vitiello C. A replication attempt of stereotype susceptibility (Shih, Pittinsky, & Ambady, 1999). Soc Psychol. 2014;45: 194–198. doi: 10.1027/1864-9335/a000184

[5] Moon A, Roeder SS. A secondary replication attempt of stereotype susceptibility (Shih, Pittinsky, & Ambady, 1999). Soc Psychol. 2014;45: 199–201. doi: 10.1027/1864-9335/a000193

[6] Nadler JT, Clark MH. Stereotype threat: A meta-analysis comparing African Americans to Hispanic Americans. J Appl Soc Psychol. 2011;41: 872–890. doi: 10.1111/j.1559-1816.2011.00739.x

[7] Sackett PR, Hardison CM, Cullen MJ. On interpreting stereotype threat as accounting for African American-White differences on cognitive tests. Am Psychol. 2004;59: 7–13. doi: 10.1037/0003-066X.59.1.7 14736315

[8] Liu S, Liu P, Wang M, Zhang B. Effectiveness of stereotype threat interventions: A meta-analytic review. J Appl Psychol. 2021;106: 921–949. doi: 10.1037/apl0000770 32772526

[9] Steele CM. A threat in the air: How stereotypes shape intellectual identity and performance. Am Psychol. 1997;52: 613–629. doi: 10.1037/0003-066X.52.6.613 9174398

[10] Nisbett RE, Aronson J, Blair C, Dickens W, Flynn J, Halpern DF et al. Intelligence: New findings and theoretical developments. Am Psychol. 2012;67: 130–159. doi: 10.1037/a0026699 22233090

[11] Spencer SJ, Logel C, Davies PG. Stereotype threat. Annu Rev Psychol. 2016;67: 415–437. doi: 10.1146/annurev-psych-073115-103235 26361054

[12] Picho-Kiroga K, Turnbull A, Rodriguez-Leahy A. Stereotype threat and its problems: Theory misspecification in research, consequences, and remedies. J Adv Acad. 2021;32: 231–264. doi: 10.1177/1932202x20986161

[13] Kaufman SB. Ungifted: Intelligence redefined. New York: Basic Books; 2013.

[14] Walton GM, Spencer SJ. Latent ability: Grades and test scores systematically underestimate the intellectual ability of negatively stereotyped students. Psychol Sci. 2009;20: 1132–1139. doi: 10.1111/j.1467-9280.2009.02417.x 19656335

[15] Worrell FC, Dixson DD. Recruiting and retaining underrepresented gifted students. In Pfeiffer SI, editor. Handbook of giftedness in children: Psychoeducational theory, research, and best practices. Cham, Switzerland: Springer International Publishing; 2018. pp. 209–226.

[16] Cross TL, Cross JR. Challenging an idea whose time has gone. Roeper Rev. 2017;39: 191–194. doi: 10.1080/02783193.2017.1319000

[17] Pennington CR, Heim D, Levy AR, Larkin DT. Twenty years of stereotype threat research: A review of psychological mediators. PLoS One. 2016;11: e0146487. doi: 10.1371/journal.pone.0146487 26752551 PMC4713435

[18] Smittick AL. The in-between: A meta-analytic investigation of stereotype threat effects on mediators of the stereotype threat-performance relationship. PhD Dissertation, Texas A&M University. 2019. https://oaktrust.library.tamu.edu/handle/1969.1/188801.

[19] Ferguson CJ, Brown JM, Torres AV. Education or indoctrination? The accuracy of introductory psychology textbooks in covering controversial topics and urban legends about psychology. Curr Psychol. 2018;37: 574–582. doi: 10.1007/s12144-016-9539-7

[20] Lewis NA, Michalak NM. Has stereotype threat dissipated over time? A cross-temporal meta-analysis. PsyArXiv:w4ta2 [Preprint]. 2019 [cited 2024 January 4].

[21] Hollis-Sawyer LA, Sawyer TP. Potential stereotype threat and face validity effects on cognitive-based test performance in the classroom. Educ Psychol. 2008;28: 291–304. doi: 10.1080/01443410701532313

[22] Shewach OR, Sackett PR, Quint S. Stereotype threat effects in settings with features likely versus unlikely in operational test settings: A meta-analysis. J Appl Psychol. 2019;104: 1514–1534. doi: 10.1037/apl0000420 31094540

[23] Meehl PE. Why summaries of research on psychological theories are often uninterpretable. Psychol Rep. 1990;66: 195–244. doi: 10.2466/pr0.1990.66.1.195

[24] Ganley CM, Mingle LA, Ryan AM, Ryan K, Vasilyeva M, Perry M. An examination of stereotype threat effects on girls’ mathematics performance. Dev Psychol. 2013;49: 1886–1897. doi: 10.1037/a0031412 23356523

[25] Kellow JT, Jones BD. The effects of stereotypes on the achievement gap: Reexamining the academic performance of African American high school students. J Black Psychol. 2008;34: 94–120. doi: 10.1177/0095798407310537

[26] Sabatine, E. Blooming where they’re planted: Closing cognitive achievement gaps with non-cognitive skills. PhD Dissertation, University of North Carolina. 2019. https://cdr.lib.unc.edu/concern/dissertations/0c483p86h.

[27] Sunny CE, Taasoobshirazi G, Clark L, Marchand G. Stereotype threat and gender differences in chemistry. Instr Sci. 2017;45: 157–175. doi: 10.1007/s11251-016-9395-8

[28] Priest R, Grievie A, Zhou Y, Tomeh D, Sackett PR. Stereotype lift and stereotype threat effects on subgroup mean differences for cognitive tests: A meta-analysis of adult samples. J Appl Psychol. in press. doi: 10.1037/apl0001185 38421748

[29] Nguyen H-HD, Ryan AM. Does stereotype threat affect test performance of minorities and women? A meta-analysis of experimental evidence. J Appl Psychol. 2008;96: 1314–1334. doi: 10.1037/a0012702 19025250

[30] Wax AL. Stereotype threat: A case of overclaim syndrome? In Sommers CH, editor. The science on women and science. Washington, DC: AIE Press; 2009. pp. 132–169.

[31] Warne RT. No strong evidence of stereotype threat in females: A reassessment of the Picho-Kiroga et al. (2021) meta-analysis. J Adv Acad. 2022;33: 171–186. doi: 10.1177/1932202x211061517

[32] Flore PC, Wicherts JM. Does stereotype threat influence performance of girls in stereotyped domains? A meta-analysis. J Sch. Psychol. 2015;53: 25–44. doi: 10.1016/j.jsp.2014.10.002 25636259

[33] Zigerell LJ. Potential publication bias in the stereotype threat literature: Comment on Nguyen and Ryan (2008). J Appl Psychol. 2017;102: 1159–1168. doi: 10.1037/apl0000188 28795832

[34] Stoet G, Geary DC. Can stereotype threat explain the gender gap in mathematics performance and achievement? Rev Gen Psychol. 2012;16: 93–102. doi: 10.1037/a0026617

[35] Open Science Collaboration. Estimating the reproducibility of psychological science. Science. 2015;349: aac4716. doi: 10.1126/science.aac4716 26315443

[36] Soto CJ. How replicable are links between personality traits and consequential life outcomes? The life outcomes of personality replication project. Psychol Sci. 2019;30: 711–727. doi: 10.1177/0956797619831612 30950321

[37] O’Donnell M, Nelson LD, Ackermann E, Aczel B, Akhtar A, Aldrovandi S. et al. Registered replication report: Dijksterhuis and van Knippenberg (1998). Perspect Psychol Sci. 2018;13: 268–294. doi: 10.1177/1745691618755704 29463182

[38] Rohrer D, Pashler H, Harris CR. Discrepant data and improbable results: An examination of Vohs, Mead, and Goode (2006). Basic Appl Soc Psych. 2019;41: 263–271. doi: 10.1080/01973533.2019.1624965

[39] Schimmack, U. Social psychology textbook audit: Stereotype threat. 2019 Jan 2 [cited 2023 May 14]. https://replicationindex.com/2019/01/02/social-psychology-textbook-audit-stereotype-threat/.

[40] Simmons JP, Nelson LD, Simonsohn U. False-positive psychology: Undisclosed flexibility in data collection and analysis allows presenting anything as significant. Psychol Sci. 2011;22: 1359–1366. doi: 10.1177/0956797611417632 22006061

[41] Simonsohn U, Nelson LD, Simmons JP. *P*-curve: a key to the file-drawer. J Exp Psychol. 2014;143: 534–547. doi: 10.1037/a0033242 23855496

[42] Carter EC, Schönbrodt FD, Gervais WM, Hilgard J. Correcting for bias in psychology: A comparison of meta-analytic methods. Adv Meth Pract Psychol Sci. 2019;2: 115–144. doi: 10.1177/2515245919847196

[43] Renkewitz F, Keiner M. How to detect publication bias in psychological research: A comparative evaluation of six statistical methods. Z fur Psychol. 2019;227: 261–279. doi: 10.1027/2151-2604/a000386

[44] Warne RT. In the know: Debunking 35 myths about human intelligence. New York: Cambridge University Press; 2020.

[45] Lee JJ. Review of Intelligence and how to get it: Why schools and cultures count. Pers Individ Diff. 2010;48: 247–255. doi: 10.1016/j.paid.2009.09.015

[46] Appel M, Weber S, Kronberger N. The influence of stereotype threat on immigrants: Review and meta-analysis. Front Psychol. 2015;6: 900. doi: 10.3389/fpsyg.2015.00900 26217256 PMC4495554

[47] Moore GW, Slate JR. Who’s taking the Advanced Placement courses and how are they doing: A statewide two-year study. High Sch J. 2008;92: 56–67. doi: 10.1353/hsj.0.0013

[48] Murray C. Facing reality: Two truths about race in America. New York: Encounter Books; 2021.

[49] Yoon SY, Gentry M. Racial and ethnic representation in gifted programs: Current status of and implications for gifted Asian American students. Gift Child Q. 2009;53: 121–136. doi: 10.1177/0016986208330564

[50] Kaplan JM. Race, IQ, and the search for statistical signals associated with so-called “X”-factors: environments, racism, and the “hereditarian hypothesis”. Biol Philos. 2015;30: 1–17. doi: 10.1007/s10539-014-9428-0

[51] Ogbu JU. Cultural amplifiers of intelligence: IQ and minority status in cross-cultural perspective. In Fish JM, editor. Race and intelligence: Separating science from myth. Mahwah, NJ: Lawrence Erlbaum Associates; 2002. pp. 241–278.

[52] Shamseer L, Moher D, Clarke M, Ghersi D, Liberati A et al. (2015). Preferred reporting items for systematic review and meta-analysis protocols (PRISMA-P) 2015: Elaboration and explanation. BMJ-Brit Med J. 2015;349: doi: 10.1136/bmj.g7647 25555855

[53] van den Akker OR, Peters G-JY, Bakker CJ, Carlsson R, Coles NA, Corker KS, Feldman G, Moreau D, Nordström T, Pickering JS, Riegelman A, Topor MK, van Veggel N, Yeung SK, Call M, Mellor DT, Pfeiffer N. Increasing the transparency of systematic reviews: presenting a generalized registration form. Syst Rev. 2023;12: 170. doi: 10.1186/s13643-023-02281-7 37736736 PMC10514995

[54] Protzko J, Aronson J. Context moderates affirmation effects on the ethnic achievement gap. Soc Psychol Personal Sci. 2016;7: 500–507. doi: 10.1177/1948550616646426

[55] Enders CK. Applied missing data analysis. Guilford Publications; 2002.

[56] Viechtbauer W. Conducting meta-analyses in R with the metafor package. J Stat Softw. 2010;36: 1–48. doi: 10.18637/jss.v036.i03

[57] Higgins JPT, Thompson SG, Deeks JJ, Altman DG. Measuring inconsistency in meta-analyses. BMJ. 2003;327:557–560. doi: 10.1136/bmj.327.7414.557 12958120 PMC192859

[58] Cochran WG. The combination of estimates from different experiments. Biom. 1954;10: 101–129 doi: 10.2307/3001666

[59] Schmidt FL. Statistical and measurement pitfalls in the use of meta-regression in meta-analysis. Career Dev Int. 2017;22: 469–476. doi: 10.1108/CDI-08-2017-0136

[60] Walton GM, Cohen GL. Stereotype lift. J Exp Soc Psychol. 2003;39: 456–467. doi: 10.1016/S0022-1031(03)00019-2

[61] Warne RT. Statistics for the social sciences: A general linear model approach. 2nd ed. New York: Cambridge University Press; 2021.

[62] Ioannidis J. P. A., & Trikalinos T. A. An exploratory test for an excess of significant findings. Clin Trials. 2007;4: 245–253. doi: 10.1177/1740774507079441 17715249

[63] Simonsohn U, Nelson LD, Simmons JP. *p*-curve and effect size: Correcting for publication bias using only significant results. Perspect Psychol Sci. 2014;9: 666–681. doi: 10.1177/1745691614553988 26186117

[64] Bartoš F, Schimmack U. zcurve: an R package for fitting z-curves. 2020. https://CRAN.R-project.org/package=zcurve

[65] Bartoš F, Schimmack U (2022). *Z*-curve 2.0: Estimating replication rates and discovery rates. Meta-Psychology. 2022;6. doi: 10.15626.MP.2021.2720

[66] van Aert RCM, Wicherts JM, van Assen MALM. (2016). Conducting meta-analyses based on *p* values: Reservations and recommendations for applying *p*-uniform and *p*-curve. Perspect Psychol Sci. 2016;11: 713–729. doi: 10.1177/1745691616650874 27694466 PMC5117126

[67] Nakagawa S, Lagisz M, Jennions MD, Koricheva J, Noble DWA, Parker TH, Sánchez-Tójar A, Yany Y, O’Dea RE. Methods for testing publication bias in ecological and evolutionary meta-analyses. Methods Ecol Evol. 2022;13: 4–21. doi: 10.1111/2041-210X.13724

[68] Sterne JAC, Gavaghan D, Egger M. Publication and related bias in meta-analysis: Power of statistical tests and prevalence in the literature. J Clin Epidemiol. 2000;53: 1119–1129. doi: 10.1016/s0895-4356(00)00242-0 11106885

[69] Macnamara BN, Burgoyne AP. Do growth mindset interventions impact students’ academic achievement? A systematic review and meta-analysis with recommendations for best practices. 2023;149: 133–173. doi: 10.1037/bul0000352 36326645

