# Supplementary material for: Protocol for a meta-analysis of stereotype threat in African Americans
Source: PLoS One. 2024 Jul 24;19(7):e0306030. doi: 10.1371/journal.pone.0306030 (PMC11268653; doi:10.1371/journal.pone.0306030)
Supplement: S1 Appendix — (DOCX) [file pone.0306030.s002.docx]

**Appendix A**

Below are the variables that will be coded in the meta-analysis. Note that each design has its own section. Potential covariates are shaded in grey.

**Within-subjects design (pre-test scores as control group)**

| Column | Label | Definition | Values |
| --- | --- | --- | --- |
| Document/Study Information | | | |
| A | Document No. |  |  |
| B | Study No. |  |  |
| C | Effect Size No. |  |  |
| D | Author(s) |  |  |
| E | Year |  |  |
| F | Author label for study | Appropriate if multiple studies are reported in the same article/manuscript. |  |
| G | DOI link or URL |  |  |
| H | Document Title |  |  |
| I | Type of Document |  | 0 = Published article, 1 = Pre-print, 2 = Dissertation/Thesis, 3 = Other Public Report, 4 = Uncirculated document/data |
| J | Sample description | Description of sample members |  |
| K | Pre-registered? | Describes whether the study was pre-registered before data collection. | 0 = No, 1 = Yes |
| L | URL of pre-registration |  |  |
| M | Pre-registration recorded before data collection? |  | 0 = No, 1 = Yes |
| N | Pre-registration followed | Final study methodology must be close to the description in the pre-registration; any departures must be minor and/or explained and justified in the report. | 0 = No, 1 = Yes |
| O | Replication? | Author(s) described the study as a replication of a specific earlier stereotype threat study | 0 = No, 1 = Yes |
| P | Citation of study being replicated |  |  |
|  | Setting | | |
| Q | Setting |  | 0 = Laboratory or artificial setting, 1 = Subjects’ school or classroom, 2 = Clinic, 3 = Home, 4 = Other |
| R | Individual or group setting | Records whether subjects were exposed to the stereotype threat intervention individually or in a group setting | 0 = Individually, 1 = Group |
| S | Average proportion of subject group that was Black | Proportion of people in the group exposed to the stereotype intervention who were African American |  |
| Independent Variable Information | | | |
| T | Stereotype threat manipulation description | Authors’ description of the stereotype threat intervention |  |
| U | Type of stereotype threat intervention | Stereotype threat triggers are designed to create the stereotype threat phenomenon in sample members. Mitigation interventions are designed to eliminate or reduce the impact of stereotype threat that is presumed to already be present. | 0 = Trigger, 1 = Mitigation, 2 = Other |
| V | Stereotype threat intervention description | Authors’ description of the stereotype threat intervention |  |
| W | Time lag between intervention and post-test | Recorded in days (if same day, report 0) |  |
| X | Manipulation check |  | 0 = None mentioned, 1 = Present and unsuccessful, 2 = Present and successful |
| Intervening, Moderator, and Mediator Variable Information | | | |
| Y | Level of self-identification with construct (as reported by authors) | If authors do not report percentages of people who self-identify with the construct, then the proportion will be estimated “Some,” “Most,” or “All” by using the mean and standard deviation to estimate (under a normal distribution) the proportion of sample members who score at or above the midpoint of the self-identification scale. | Proportion of sample members who report self-identifying with construct. |
| Z | Task difficulty (as reported by authors) | If authors do not report exact percentages, but do label the task as “easy,” “medium difficulty,” or “hard,” (or similar terminology), then this will be coded. | 0 = Easy (mean score of 80% or higher) , 1 = Medium (mean score of 51-79%), 2 = Hard (mean score of 0-50%) |
| AA | Stereotype awareness (as reported by authors) | If awareness is reported via a rating scale, then “awareness” will be defined as scoring above the midpoint on the scale. Percentages of sample members above the midpoint may be estimated by using the mean and standard deviation (under a normal distribution). | Proportion of sample members who report being aware of the stereotype |
| AB | Stereotype belief (as reported by authors) | If belief/endorsement is reported via a rating scale, then “belief” will be defined as scoring above the midpoint on the scale. Percentages of sample members above the midpoint may be estimated by using the mean and standard deviation (under a normal distribution). | Proportion of sample members who endorse or state that they believe the stereotype to be true. |
| AC | Anxiety (as reported by authors) | Physiological measures of anxiety recorded during the task are preferred and will take precedence over self-reports.  If anxiety is reported via a rating scale, then “anxiety” will be defined as scoring above the midpoint on the scale. Percentages of sample members above the midpoint may be estimated by using the mean and standard deviation (under a normal distribution). | Proportion of sample members who exhibit or report having anxiety about the task. |
| AD | Average level of motivation for participants (as reported by authors) | Scale will be divided into thirds. A mean or median in the lowest third of the motivation scale will be defined as “low motivation.” A mean or median in the middle third of the scale will be defined as “medium motivation.” A mean in the top third of the scale will be defined as “high motivation.” | 0 = Low, 1 = Medium, 2 = High |
| Dependent Variable Information | | | |
| AE | Name of measure of the dependent variable |  |  |
| AF | Construct measured by the dependent variable |  | 0 = Specific academic achievement, 1 = General academic achievement, 2 = Specific academic aptitude, 3 = Intelligence or IQ, 4 = Other |
| AG | Authors’ name for the construct measured by the dependent variable |  |  |
| AH | Dependent variable type |  | 0 = Subjective rating, 1 = Experimenter-created test score, 2 = Single course grade, 3 = Multiple course grades (e.g., GPA), 4 = Standardized test altered by experimenter, 5 = Standardized test score for unaltered test, 6 = Other |
| AI | Description of “Other” dependent variable type |  |  |
| AJ | Test scorer status |  | 0 = Objective, 1 = Blind, 2 = Non-blind |
| AK | Stakes of dependent variable |  | 0 = No/low stakes, 1 = High stakes for course grade, 2 = Non-grade incentive for high performance |
| Sample Characteristics | | | |
| AL | Sample type |  | 0 = Community, 1 = College or university students, 2 = K-12 students, 3 = Other |
| AM | Description of “Other” sample type |  |  |
| AN | Median or mean sample socioeconomic status |  | 0 = Low, 1 = Medium or middle class, 2 = High |
| AO | Population demographics |  | 0 = African Americans are a minority of community or school, 1 = African Americans are a majority of community or school, 2 = Academic program designed for African American students, 3 = Other |
| AP | Description of “other” population demographics |  |  |
| AQ | Academic selectivity of sample |  | 0 = Academically struggling group, 1 = Academically typical/mixed/general group, 2 = Academically elite group |
| AR | Sample size | Not counting excluded participants. |  |
| AS | Number of males in sample |  |  |
| AT | Number of females in sample |  |  |
| Results | | | |
| AU | Pre-test mean |  |  |
| AV | Pre-test SD |  |  |
| AW | Post-test mean |  |  |
| AX | Post-test SD |  |  |
| AY | Reported effect size |  |  |
| AZ | Reported effect size value |  |  |
| BA | Reported *p*-value |  |  |
| BB | Reported *p*-value > .05 interpreted to support stereotype threat theory? |  | 0 = No, 1 = Yes |
| BC | Recalculated effect size |  |  |
| BD | Recalculated effect size value | Using pre-test SD in the denominator. |  |
| BE | Denominator used to calculate effect size | Authors’ choice of denominator. |  |
| BF | Recalculated *p*-value |  |  |
| Statistical power | | | |
| BG | Authors reported statistical power? | Statistical power must be a priori power (not post hoc power). | 0 = No, 1 = Yes |
| BH | Authors’ a priori effect size value used to calculate statistical power |  |  |
| BI | Authors’ reported a priori statistical power |  |  |
| BJ | Recalculated a priori statistical power | Effect size for this calculation is *d* = .20. |  |
| Data/Sample Member Exclusion | | | |
| BK | Were sample members excluded? |  | 0 = No, 1 = Yes |
| BL | Reason(s) for sample member exclusion |  |  |
| BM | Were DV(s) excluded? |  | 0 = No, 1 = Yes |
| BN | Names of DV(s) excluded |  |  |
| BO | Reason(s) that DV(s) were excluded |  |  |
| Covariate Control | | | |
| BP | Researcher controlled for covariates or confounding variables | This does *not* include using random assignment to control for confounding variables. | 0 = No, 1 = Yes |
| BQ | Primary method for controlling for covariates or confounding variables |  | 0 = ANCOVA, 1 = Matching, 2 = Regression, 3 = Other |
| BR | Description of “Other” primary method for controlling for covariates or confounding variables |  |  |
| BS | Multiple methods for controlling for covariates or confounding variables present? |  | 0 = No, 1 = Yes |
| BT | Names of other methods used to control for covariates or confounding variables | This does not include the primary method. |  |
| Miscellaneous Notes | | | |
| BU | Notes |  |  |

**Between-groups design (comparing groups of African Americans only)**

| Column | Label | Definition | Values |
| --- | --- | --- | --- |
| Document/Study Information | | | |
| A | Document No. |  |  |
| B | Study No. |  |  |
| C | Effect Size No. |  |  |
| D | Author(s) |  |  |
| E | Year |  |  |
| F | Author label for study | Appropriate if multiple studies are reported in the same article/manuscript. |  |
| G | DOI link or URL |  |  |
| H | Document Title |  |  |
| I | Type of Document |  | 0 = Published article, 1 = Pre-print, 2 = Dissertation/Thesis, 3 = Other Public Report, 4 = Uncirculated document/data |
| J | Sample description | Description of sample members |  |
| K | Pre-registered? | Describes whether the study was pre-registered before data collection | 0 = No, 1 = Yes |
| L | URL of pre-registration |  |  |
| M | Pre-registration recorded before data collection? |  | 0 = No, 1 = Yes |
| N | Pre-registration followed | Final study methodology must be close to the description in the pre-registration; any departures must be minor and/or explained and justified in the report. | 0 = No, 1 = Yes |
| O | Replication? | Author(s) described the study as a replication of a specific earlier stereotype threat study | 0 = No, 1 = Yes |
| P | Citation of study being replicated |  |  |
|  | Setting | | |
| Q | Setting |  | 0 = Laboratory or artificial setting, 1 = Subjects’ school or classroom, 2 = Clinic, 3 = Home, 4 = Other |
| R | Individual or group setting | Records whether subjects were exposed to the stereotype threat intervention individually or in a group setting | 0 = Individually, 1 = Group |
| S | Average proportion of subject group that was Black | Proportion of people in the group exposed to the stereotype intervention who were African American |  |
| Independent Variable Information | | | |
| T | Random assignment level |  | 0 = Individual, 1 = Group |
| U | Type of stereotype threat intervention | Stereotype threat triggers are designed to create the stereotype threat phenomenon in sample members. Mitigation interventions are designed to eliminate or reduce the impact of stereotype threat that is presumed to already be present. | 0 = Trigger, 1 = Mitigation, 2 = Other |
| V | Stereotype threat intervention description | Authors’ description of the stereotype threat intervention |  |
| W | Time lag between intervention and post-test | Recorded in days (if same day, report 0) |  |
| X | Manipulation check |  | 0 = None mentioned, 1 = Present and unsuccessful, 2 = Present and successful |
| Y | Control group experience | To be an “active control” experience, control group members must experience a placebo intervention of similar duration and delivery as the experimental group’s experience. Otherwise, the experience should be considered “passive control.” | 0 = Passive control (i.e., typical academic/testing experience), 1 = Active control |
| Intervening, Moderator, and Mediator Variable Information | | | |
| Z | Level of self-identification with construct (as reported by authors) | If authors do not report percentages of people who self-identify with the construct, then the proportion will be estimated “Some,” “Most,” or “All” by using the mean and standard deviation to estimate (under a normal distribution) the proportion of sample members who score at or above the midpoint of the self-identification scale. | Proportion of sample members who report self-identifying with construct. |
| AA | Task difficulty (as reported by authors) | If authors do not report exact percentages, but do label the task as “easy,” “medium difficulty,” or “hard,” (or similar terminology), then this will be coded. | 0 = Easy (mean score of 80% or higher) , 1 = Medium (mean score of 51-79%), 2 = Hard (mean score of 0-50%) |
| AB | Stereotype awareness (as reported by authors) | If awareness is reported via a rating scale, then “awareness” will be defined as scoring above the midpoint on the scale. Percentages of sample members above the midpoint may be estimated by using the mean and standard deviation (under a normal distribution). | Proportion of sample members who report being aware of the stereotype |
| AC | Stereotype belief (as reported by authors) | If belief/endorsement is reported via a rating scale, then “belief” will be defined as scoring above the midpoint on the scale. Percentages of sample members above the midpoint may be estimated by using the mean and standard deviation (under a normal distribution). | Proportion of sample members who endorse or state that they believe the stereotype to be true. |
| AD | Anxiety (as reported by authors) | Physiological measures of anxiety recorded during the task are preferred and will take precedence over self-reports.  If anxiety is reported via a rating scale, then “anxiety” will be defined as scoring above the midpoint on the scale. Percentages of sample members above the midpoint may be estimated by using the mean and standard deviation (under a normal distribution). | Proportion of sample members who exhibit or report having anxiety about the task. |
| AE | Average level of motivation for participants (as reported by authors) | Scale will be divided into thirds. A mean or median in the lowest third of the motivation scale will be defined as “low motivation.” A mean or median in the middle third of the scale will be defined as “medium motivation.” A mean in the top third of the scale will be defined as “high motivation.” | 0 = Low, 1 = Medium, 2 = High |
| Dependent Variable Information | | | |
| AF | Name of measure of the dependent variable | DV should be a measure of academic or cognitive aptitude or achievement. |  |
| AG | Construct measured by the dependent variable |  | 0 = Specific academic achievement, 1 = General academic achievement, 2 = Specific academic aptitude, 3 = Intelligence or IQ, 4 = Other |
| AH | Authors’ name for the construct measured by the dependent variable |  |  |
| AI | Dependent variable type |  | 0 = Subjective rating, 1 = Experimenter-created test score, 2 = Single course grade, 3 = Multiple course grades (e.g., GPA), 4 = Standardized test altered by experimenter, 5 = Standardized test score for unaltered test, 6 = Other |
| AJ | Description of “Other” dependent variable type |  |  |
| AK | Test scorer status |  | 0 = Objective, 1 = Blind, 2 = Non-blind |
| AL | Stakes of dependent variable |  | 0 = No/low stakes, 1 = High stakes for course grade, 2 = Non-grade incentive for high performance |
| Sample Characteristics | | | |
| AM | Sample type |  | 0 = Community, 1 = College or university students, 2 = K-12 students, 3 = Other |
| AN | Description of “Other” sample type |  |  |
| AO | Median or mean sample socioeconomic status |  | 0 = Low, 1 = Medium or middle class, 2 = High |
| AP | Population demographics |  | 0 = African Americans are a minority of community or school, 1 = African Americans are a majority of community or school, 2 = Academic program designed for African American students, 3 = Other |
| AQ | Description of “other” population demographics |  |  |
| AR | Academic selectivity of sample |  | 0 = Academically struggling group, 1 = Academically typical/mixed/general group, 2 = Academically elite group |
| AS | Total sample size | Not counting excluded participants. |  |
| AT | Sample size of intervention group | Not counting excluded participants. |  |
| AU | Sample size of control group | Not counting excluded participants. |  |
| AV | Total number of males in sample |  |  |
| AW | Total number of females in sample |  |  |
| Results | | | |
| AX | Pre-test mean for intervention group |  |  |
| AY | Pre-test SD for intervention group |  |  |
| AZ | Post-test mean for intervention group |  |  |
| BA | Post-test SD for intervention group |  |  |
| BB | Pre-test mean for control group |  |  |
| BC | Pre-test SD for control group |  |  |
| BD | Post-test mean for control group |  |  |
| BE | Post-test SD for control group |  |  |
| BF | Reported effect size | Post-test dependent variable only |  |
| BG | Reported effect size value | Post-test dependent variable only |  |
| BH | Reported *p*-value | Post-test dependent variable only |  |
| BI | Reported *p*-value > .05 interpreted to support stereotype threat theory? |  | 0 = No, 1 = Yes |
| BJ | Recalculated effect size | Post-test dependent variable only |  |
| BK | Recalculated effect size value | Using pooled SD in the denominator. |  |
| BL | Denominator used to calculate effect size | Authors’ choice of denominator. |  |
| BM | Recalculated *p*-value | Post-test dependent variable only |  |
| Statistical power | | | |
| BN | Authors reported statistical power? | Statistical power must be a priori power (not post hoc power). | 0 = No, 1 = Yes |
| BO | Authors’ a priori effect size value used to calculate statistical power |  |  |
| BP | Authors’ reported a priori statistical power |  |  |
| BQ | Recalculated a priori statistical power | Effect size for this calculation is d = .20. |  |
| Data/Sample Member Exclusion | | | |
| BR | Were sample members excluded? |  | 0 = No, 1 = Yes |
| BS | Reason(s) for sample member exclusion |  |  |
| BT | Were DV(s) excluded? |  | 0 = No, 1 = Yes |
| BU | Names of DV(s) excluded |  |  |
| BV | Reason(s) that DV(s) were excluded |  |  |
| Covariate Control | | | |
| BW | Researcher controlled for covariates or confounding variables | This does *not* include using random assignment to control for confounding variables. | 0 = No, 1 = Yes |
| BX | Primary method for controlling for covariates or confounding variables |  | 0 = ANCOVA, 1 = Matching, 2 = Regression, 3 = Other |
| BY | Description of “Other” primary method for controlling for covariates or confounding variables |  |  |
| BZ | Multiple methods for controlling for covariates or confounding variables present? |  | 0 = No, 1 = Yes |
| CA | Names of other methods used to control for covariates or confounding variables | This does not include the primary method. |  |
| Miscellaneous Notes | | | |
| CB | Notes |  |  |

**Between-groups design (comparing groups of African Americans and non-stereotyped individuals)**

| Column | Label | Definition | Values |
| --- | --- | --- | --- |
| Document/Study Information | | | |
| A | Document No. |  |  |
| B | Study No. |  |  |
| C | Effect Size No. |  |  |
| D | Author(s) |  |  |
| E | Year |  |  |
| F | Author label for study | Appropriate if multiple studies are reported in the same article/manuscript. |  |
| G | DOI link or URL |  |  |
| H | Document Title |  |  |
| I | Type of Document |  | 0 = Published article, 1 = Pre-print, 2 = Dissertation/Thesis, 3 = Other Public Report, 4 = Uncirculated document/data |
| J | Sample description | Description of sample members |  |
| K | Pre-registered? | Describes whether the study was pre-registered before data collection | 0 = No, 1 = Yes |
| L | URL of pre-registration |  |  |
| M | Pre-registration recorded before data collection? |  | 0 = No, 1 = Yes |
| N | Pre-registration followed | Final study methodology must be close to the description in the pre-registration; any departures must be minor and/or explained and justified in the report. | 0 = No, 1 = Yes |
| O | Replication? | Author(s) described the study as a replication of a specific earlier stereotype threat study | 0 = No, 1 = Yes |
| P | Citation of study being replicated |  |  |
|  | Setting | | |
| Q | Setting |  | 0 = Laboratory or artificial setting, 1 = Subjects’ school or classroom, 2 = Clinic, 3 = Home, 4 = Other |
| R | Individual or group setting | Records whether subjects were exposed to the stereotype threat intervention individually or in a group setting | 0 = Individually, 1 = Group |
| S | Average proportion of subject group that was Black | Proportion of people in the group exposed to the stereotype intervention who were African American |  |
| Independent Variable Information | | | |
| T | Random assignment level |  | 0 = Individual, 1 = Group |
| U | Type of stereotype threat intervention | Stereotype threat triggers are designed to create the stereotype threat phenomenon in sample members. Mitigation interventions are designed to eliminate or reduce the impact of stereotype threat that is presumed to already be present. | 0 = Trigger, 1 = Mitigation, 2 = Other |
| V | Stereotype threat intervention description | Authors’ description of the stereotype threat intervention |  |
| W | Time lag between intervention and post-test | Recorded in days (if same day, report 0) |  |
| X | Manipulation check |  | 0 = None mentioned, 1 = Present and unsuccessful, 2 = Present and successful |
| Y | Control group experience | To be an “active control” experience, control group members must experience a placebo intervention of similar duration and delivery as the experimental group’s experience. Otherwise, the experience should be considered “passive control.” | 0 = Passive control (i.e., typical academic/testing experience), 1 = Active control |
| Intervening, Moderator, and Mediator Variable Information | | | |
| Z | Level of self-identification with construct (as reported by authors) | Note: For studies/designs that have non-Black participants, this should be the level of self-identification with the construct among African Americans.  If authors do not report percentages of people who self-identify with the construct, then the proportion will be estimated “Some,” “Most,” or “All” by using the mean and standard deviation to estimate (under a normal distribution) the proportion of sample members who score at or above the midpoint of the self-identification scale. | Proportion of sample members who report self-identifying with construct. |
| AA | Task difficulty (as reported by authors) | Note: For studies/designs that have non-Black participants, this should be the difficulty of the task for African Americans.  If authors do not report exact percentages, but do label the task as “easy,” “medium difficulty,” or “hard,” (or similar terminology), then this will be coded. | 0 = Easy (mean score of 80% or higher) , 1 = Medium (mean score of 51-79%), 2 = Hard (mean score of 0-50%) |
| AB | Stereotype awareness (as reported by authors) | Note: For studies/designs that have non-Black participants, this should be the proportion of African Americans who are aware of the stereotype.  If authors do not report exact percentages, but do label the task as “easy,” “medium difficulty,” or “hard,” (or similar terminology), then this will be coded. | Proportion of sample members who report being aware of the stereotype |
| AC | Stereotype belief (as reported by authors) | Note: For studies/designs that have non-Black participants, this should be the proportion of African Americans who believe the stereotype.  If authors do not report exact percentages, but do label the task as “easy,” “medium difficulty,” or “hard,” (or similar terminology), then this will be coded. | Proportion of sample members who endorse or state that they believe the stereotype to be true. |
| AD | Anxiety (as reported by authors) | Note: For studies/designs that have non-Black participants, this should be the proportion of African Americans who demonstrate or report anxiety.  Physiological measures of anxiety recorded during the task are preferred and will take precedence over self-reports.  If anxiety is reported via a rating scale, then “anxiety” will be defined as scoring above the midpoint on the scale. Percentages of sample members above the midpoint may be estimated by using the mean and standard deviation (under a normal distribution). | Proportion of sample members who exhibit or report having anxiety about the task. |
| AE | Average level of motivation for participants (as reported by authors) | Note: For studies/designs that have non-Black participants, this should be the average level of motivation in African Americans.  Physiological measures of anxiety recorded during the task are preferred and will take precedence over self-reports.  If anxiety is reported via a rating scale, then “anxiety” will be defined as scoring above the midpoint on the scale. Percentages of sample members above the midpoint may be estimated by using the mean and standard deviation (under a normal distribution). | 0 = Low, 1 = Medium, 2 = High |
| Dependent Variable Information | | | |
| AF | Name of measure of the dependent variable | DV should be a measure of academic or cognitive aptitude or achievement. |  |
| AG | Construct measured by the dependent variable |  | 0 = Specific academic achievement, 1 = General academic achievement, 2 = Specific academic aptitude, 3 = Intelligence or IQ, 4 = Other |
| AH | Authors’ name for the construct measured by the dependent variable |  |  |
| AI | Dependent variable type |  | 0 = Subjective rating, 1 = Experimenter-created test score, 2 = Single course grade, 3 = Multiple course grades (e.g., GPA), 4 = Standardized test altered by experimenter, 5 = Standardized test score for unaltered test, 6 = Other |
| AJ | Description of “Other” dependent variable type |  |  |
| AK | Test scorer status |  | 0 = Objective, 1 = Blind, 2 = Non-blind |
| AL | Stakes of dependent variable |  | 0 = No/low stakes, 1 = High stakes for course grade, 2 = Non-grade incentive for high performance |
| Sample Characteristics | | | |
| AM | Sample type |  | 0 = Community, 1 = College or university students, 2 = K-12 students, 3 = Other |
| AN | Description of “Other” sample type |  |  |
| AO | Median or mean sample socioeconomic status |  | 0 = Low, 1 = Medium or middle class, 2 = High |
| AP | Population demographics |  | 0 = African Americans are a minority of community or school, 1 = African Americans are a majority of community or school, 2 = Academic program designed for African American students, 3 = Other |
| AQ | Description of “other” population demographics |  |  |
| AR | Academic selectivity of sample |  | 0 = Academically struggling group, 1 = Academically typical/mixed/general group, 2 = Academically elite group |
| AS | Non-stereotyped group | Description of the non-stereotyped group members in the study. |  |
| AT | Total Sample size | Not counting excluded participants. |  |
| AU | Sample size of Black intervention group | Not counting excluded participants. |  |
| AV | Sample size of Black control group | Not counting excluded participants. |  |
| AW | Sample size of non-stereotyped intervention group | Not counting excluded participants. |  |
| AX | Sample size of non-stereotyped control group | Not counting excluded participants. |  |
| AY | Total number of Black males in sample |  |  |
| AZ | Total number of Black females in sample |  |  |
| BA | Total number of non-stereotyped males in sample |  |  |
| BB | Total number of non-stereotyped females in sample |  |  |
| Results | | | |
| BC | Pre-test mean for Black intervention group |  |  |
| BD | Pre-test SD for Black intervention group |  |  |
| BE | Post-test mean for Black intervention group |  |  |
| BF | Post-test SD for Black intervention group |  |  |
| BG | Pre-test mean for Black control group |  |  |
| BH | Pre-test SD for Black control group |  |  |
| BI | Post-test mean for Black control group |  |  |
| BJ | Post-test SD for Black control group |  |  |
| BK | Pre-test mean for non-stereotyped intervention group |  |  |
| BL | Pre-test SD for non-stereotyped intervention group |  |  |
| BM | Post-test mean for non-stereotyped intervention group |  |  |
| BN | Post-test SD for non-stereotyped intervention group |  |  |
| BO | Pre-test mean for non-stereotyped control group |  |  |
| BP | Pre-test SD for non-stereotyped control group |  |  |
| BQ | Post-test mean for non-stereotyped control group |  |  |
| BR | Post-test SD for non-stereotyped control group |  |  |
| BS | Reported effect size | Post-test dependent variable only, comparing non-stereotyped and Black participants who were exposed to the intervention |  |
| BT | Reported effect size value | Post-test dependent variable only, comparing non-stereotyped and Black participants who were exposed to the intervention |  |
| BU | Reported *p*-value | Post-test dependent variable only, comparing non-stereotyped and Black participants who were exposed to the intervention |  |
| BV | Reported *p*-value > .05 interpreted to support stereotype threat theory? |  | 0 = No, 1 = Yes |
| BW | Recalculated effect size | Post-test dependent variable only, comparing non-stereotyped and Black participants who were exposed to the intervention |  |
| BX | Recalculated effect size value | Post-test dependent variable only, comparing non-stereotyped and Black participants who were exposed to the intervention |  |
| BY | Denominator used to calculate effect size | Post-test dependent variable only, comparing non-stereotyped and Black participants who were exposed to the intervention. Authors choice of denominator. |  |
| BZ | Recalculated *p*-value | Post-test dependent variable only, comparing non-stereotyped and Black participants who were exposed to the intervention using pooled SD as the denominator. |  |
| Statistical power | | | |
| CA | Authors reported statistical power? | Statistical power must be a priori power (not post hoc power). Effect size should be comparing non-stereotyped and Black participants who were exposed to the intervention | 0 = No, 1 = Yes |
| CB | Authors’ a priori effect size value used to calculate statistical power | Effect size should be comparing non-stereotyped and Black participants who were exposed to the intervention |  |
| CC | Authors’ reported a priori statistical power | Effect size should be comparing non-stereotyped and Black participants who were exposed to the intervention |  |
| CD | Recalculated a priori statistical power | Effect size for this calculation is d = .20. Effect size should be comparing non-stereotyped and Black participants who were exposed to the intervention |  |
| Data/Sample Member Exclusion | | | |
| CE | Were sample members excluded? |  | 0 = No, 1 = Yes |
| CF | Reason(s) for sample member exclusion |  |  |
| CG | Were DV(s) excluded? |  | 0 = No, 1 = Yes |
| CH | Names of DV(s) excluded |  |  |
| CI | Reason(s) that DV(s) were excluded |  |  |
| Covariate Control | | | |
| CJ | Researcher controlled for covariates or confounding variables | This does *not* include using random assignment to control for confounding variables between intervention and control groups. | 0 = No, 1 = Yes |
| CK | Primary method for controlling for covariates or confounding variables |  | 0 = ANCOVA, 1 = Matching, 2 = Regression, 3 = Other |
| CL | Description of “Other” primary method for controlling for covariates or confounding variables |  |  |
| CM | Multiple methods for controlling for covariates or confounding variables present? |  | 0 = No, 1 = Yes |
| CN | Names of other methods used to control for covariates or confounding variables | This does not include the primary method. |  |
| Miscellaneous Notes | | | |
| CO | Notes |  |  |

**Time x Group interaction: Between-groups design (comparing groups of African Americans only)**

| Column | Label | Definition | Values |
| --- | --- | --- | --- |
| Document/Study Information | | | |
| A | Document No. |  |  |
| B | Study No. |  |  |
| C | Effect Size No. |  |  |
| D | Author(s) |  |  |
| E | Year |  |  |
| F | Author label for study | Appropriate if multiple studies are reported in the same article/manuscript. |  |
| G | DOI link or URL |  |  |
| H | Document Title |  |  |
| I | Type of Document |  | 0 = Published article, 1 = Pre-print, 2 = Dissertation/Thesis, 3 = Other Public Report, 4 = Uncirculated document/data |
| J | Sample description | Description of sample members |  |
| K | Pre-registered? | Describes whether the study was pre-registered before data collection | 0 = No, 1 = Yes |
| L | URL of pre-registration |  |  |
| M | Pre-registration recorded before data collection? |  | 0 = No, 1 = Yes |
| N | Pre-registration followed | Final study methodology must be close to the description in the pre-registration; any departures must be minor and/or explained and justified in the report. | 0 = No, 1 = Yes |
| O | Replication? | Author(s) described the study as a replication of a specific earlier stereotype threat study | 0 = No, 1 = Yes |
| P | Citation of study being replicated |  |  |
|  | Setting | | |
| Q | Setting |  | 0 = Laboratory or artificial setting, 1 = Subjects’ school or classroom, 2 = Clinic, 3 = Home, 4 = Other |
| R | Individual or group setting | Records whether subjects were exposed to the stereotype threat intervention individually or in a group setting | 0 = Individually, 1 = Group |
| S | Average proportion of subject group that was Black | Proportion of people in the group exposed to the stereotype intervention who were African American |  |
| Independent Variable Information | | | |
| T | Random assignment level |  | 0 = Individual, 1 = Group |
| U | Type of stereotype threat intervention | Stereotype threat triggers are designed to create the stereotype threat phenomenon in sample members. Mitigation interventions are designed to eliminate or reduce the impact of stereotype threat that is presumed to already be present. | 0 = Trigger, 1 = Mitigation, 2 = Other |
| V | Stereotype threat intervention description | Authors’ description of the stereotype threat intervention |  |
| W | Time lag between intervention and post-test | Recorded in days (if same day, report 0) |  |
| X | Manipulation check |  | 0 = None mentioned, 1 = Present and unsuccessful, 2 = Present and successful |
| Y | Control group experience | To be an “active control” experience, control group members must experience a placebo intervention of similar duration and delivery as the experimental group’s experience. Otherwise, the experience should be considered “passive control.” | 0 = Passive control (i.e., typical academic/testing experience), 1 = Active control |
| Intervening, Moderator, and Mediator Variable Information | | | |
| Z | Level of self-identification with construct (as reported by authors) | If authors do not report percentages of people who self-identify with the construct, then the proportion will be estimated “Some,” “Most,” or “All” by using the mean and standard deviation to estimate (under a normal distribution) the proportion of sample members who score at or above the midpoint of the self-identification scale. | Proportion of sample members who report self-identifying with construct. |
| AA | Task difficulty (as reported by authors) | If authors do not report exact percentages, but do label the task as “easy,” “medium difficulty,” or “hard,” (or similar terminology), then this will be coded. | 0 = Easy (mean score of 80% or higher) , 1 = Medium (mean score of 51-79%), 2 = Hard (mean score of 0-50%) |
| AB | Stereotype awareness (as reported by authors) | If awareness is reported via a rating scale, then “awareness” will be defined as scoring above the midpoint on the scale. Percentages of sample members above the midpoint may be estimated by using the mean and standard deviation (under a normal distribution). | Proportion of sample members who report being aware of the stereotype |
| AC | Stereotype belief (as reported by authors) | If belief/endorsement is reported via a rating scale, then “belief” will be defined as scoring above the midpoint on the scale. Percentages of sample members above the midpoint may be estimated by using the mean and standard deviation (under a normal distribution). | Proportion of sample members who endorse or state that they believe the stereotype to be true. |
| AD | Anxiety (as reported by authors) | Physiological measures of anxiety recorded during the task are preferred and will take precedence over self-reports.  If anxiety is reported via a rating scale, then “anxiety” will be defined as scoring above the midpoint on the scale. Percentages of sample members above the midpoint may be estimated by using the mean and standard deviation (under a normal distribution). | Proportion of sample members who exhibit or report having anxiety about the task. |
| AE | Average level of motivation for participants (as reported by authors) | Scale will be divided into thirds. A mean or median in the lowest third of the motivation scale will be defined as “low motivation.” A mean or median in the middle third of the scale will be defined as “medium motivation.” A mean in the top third of the scale will be defined as “high motivation.” | 0 = Low, 1 = Medium, 2 = High |
| Dependent Variable Information | | | |
| AF | Name of measure of the dependent variable | DV should be a measure of academic or cognitive aptitude or achievement. |  |
| AG | Construct measured by the dependent variable |  | 0 = Specific academic achievement, 1 = General academic achievement, 2 = Specific academic aptitude, 3 = Intelligence or IQ, 4 = Other |
| AH | Authors’ name for the construct measured by the dependent variable |  |  |
| AI | Dependent variable type |  | 0 = Subjective rating, 1 = Experimenter-created test score, 2 = Single course grade, 3 = Multiple course grades (e.g., GPA), 4 = Standardized test altered by experimenter, 5 = Standardized test score for unaltered test, 6 = Other |
| AJ | Description of “Other” dependent variable type |  |  |
| AK | Test scorer status |  | 0 = Objective, 1 = Blind, 2 = Non-blind |
| AL | Stakes of dependent variable |  | 0 = No/low stakes, 1 = High stakes for course grade, 2 = Non-grade incentive for high performance |
| Sample Characteristics | | | |
| AM | Sample type |  | 0 = Community, 1 = College or university students, 2 = K-12 students, 3 = Other |
| AN | Description of “Other” sample type |  |  |
| AO | Median or mean sample socioeconomic status |  | 0 = Low, 1 = Medium or middle class, 2 = High |
| AP | Population demographics |  | 0 = African Americans are a minority of community or school, 1 = African Americans are a majority of community or school, 2 = Academic program designed for African American students, 3 = Other |
| AQ | Description of “other” population demographics |  |  |
| AR | Academic selectivity of sample |  | 0 = Academically struggling group, 1 = Academically typical/mixed/general group, 2 = Academically elite group |
| AS | Total sample size | Not counting excluded participants. |  |
| AT | Sample size of intervention group | Not counting excluded participants. |  |
| AU | Sample size of control group | Not counting excluded participants. |  |
| AV | Total number of males in sample |  |  |
| AW | Total number of females in sample |  |  |
| Results | | | |
| AX | Pre-test mean for intervention group |  |  |
| AY | Pre-test SD for intervention group |  |  |
| AZ | Post-test mean for intervention group |  |  |
| BA | Post-test SD for intervention group |  |  |
| BB | Pre-test mean for control group |  |  |
| BC | Pre-test SD for control group |  |  |
| BD | Post-test mean for control group |  |  |
| BE | Post-test SD for control group |  |  |
| BF | Reported interaction effect size | Post-test dependent variable only, time x group interaction |  |
| BG | Reported interaction effect size value | Post-test dependent variable only, time x group interaction |  |
| BH | Reported interaction *p*-value | Post-test dependent variable only, time x group interaction |  |
| BI | Reported interaction *p*-value > .05 interpreted to support stereotype threat theory? |  | 0 = No, 1 = Yes |
| BJ | Recalculated interaction effect size | Post-test dependent variable only, time x group interaction |  |
| BK | Recalculated interaction effect size value | Post-test dependent variable only, time x group interaction |  |
| BL | Recalculated interaction *p*-value | Post-test dependent variable only, time x group interaction |  |
| Statistical power | | | |
| BM | Authors reported statistical power for the interaction? | Statistical power must be a priori power (not post hoc power), time x group interaction. | 0 = No, 1 = Yes |
| BN | Authors’ a priori effect size value used to calculate statistical power for the interaction | Time x group interaction |  |
| BO | Authors’ reported a priori statistical power for the interaction | Time x group interaction |  |
| BP | Recalculated a priori statistical power for the interaction | Effect size for this calculation is η^2^ = .001 and a null main effect. |  |
| Data/Sample Member Exclusion | | | |
| BQ | Were sample members excluded? |  | 0 = No, 1 = Yes |
| BR | Reason(s) for sample member exclusion |  |  |
| BS | Were DV(s) excluded? |  | 0 = No, 1 = Yes |
| BT | Names of DV(s) excluded |  |  |
| BU | Reason(s) that DV(s) were excluded |  |  |
| Covariate Control | | | |
| BV | Researcher controlled for covariates or confounding variables | This does *not* include using random assignment to control for confounding variables. | 0 = No, 1 = Yes |
| BW | Primary method for controlling for covariates or confounding variables |  | 0 = ANCOVA, 1 = Matching, 2 = Regression, 3 = Other |
| BX | Description of “Other” primary method for controlling for covariates or confounding variables |  |  |
| BY | Multiple methods for controlling for covariates or confounding variables present? |  | 0 = No, 1 = Yes |
| BZ | Names of other methods used to control for covariates or confounding variables | This does not include the primary method. |  |
| Miscellaneous Notes | | | |
| CA | Notes |  |  |

**Race x group interaction: Between-groups design (comparing groups of African Americans and non-stereotyped individuals)**

| Column | Label | Definition | Values |
| --- | --- | --- | --- |
| Document/Study Information | | | |
| A | Document No. |  |  |
| B | Study No. |  |  |
| C | Effect Size No. |  |  |
| D | Author(s) |  |  |
| E | Year |  |  |
| F | Author label for study | Appropriate if multiple studies are reported in the same article/manuscript. |  |
| G | DOI link or URL |  |  |
| H | Document Title |  |  |
| I | Type of Document |  | 0 = Published article, 1 = Pre-print, 2 = Dissertation/Thesis, 3 = Other Public Report, 4 = Uncirculated document/data |
| J | Sample description | Description of sample members |  |
| K | Pre-registered? | Describes whether the study was pre-registered before data collection | 0 = No, 1 = Yes |
| L | URL of pre-registration |  |  |
| M | Pre-registration recorded before data collection? |  | 0 = No, 1 = Yes |
| N | Pre-registration followed | Final study methodology must be close to the description in the pre-registration; any departures must be minor and/or explained and justified in the report. | 0 = No, 1 = Yes |
| O | Replication? | Author(s) described the study as a replication of a specific earlier stereotype threat study | 0 = No, 1 = Yes |
| P | Citation of study being replicated |  |  |
|  | Setting | | |
| Q | Setting |  | 0 = Laboratory or artificial setting, 1 = Subjects’ school or classroom, 2 = Clinic, 3 = Home, 4 = Other |
| R | Individual or group setting | Records whether subjects were exposed to the stereotype threat intervention individually or in a group setting | 0 = Individually, 1 = Group |
| S | Average proportion of subject group that was Black | Proportion of people in the group exposed to the stereotype intervention who were African American |  |
| Independent Variable Information | | | |
| T | Random assignment level |  | 0 = Individual, 1 = Group |
| U | Type of stereotype threat intervention | Stereotype threat triggers are designed to create the stereotype threat phenomenon in sample members. Mitigation interventions are designed to eliminate or reduce the impact of stereotype threat that is presumed to already be present. | 0 = Trigger, 1 = Mitigation, 2 = Other |
| V | Stereotype threat intervention description | Authors’ description of the stereotype threat intervention |  |
| W | Time lag between intervention and post-test | Recorded in days (if same day, report 0) |  |
| X | Manipulation check |  | 0 = None mentioned, 1 = Present and unsuccessful, 2 = Present and successful |
| Y | Control group experience | To be an “active control” experience, control group members must experience a placebo intervention of similar duration and delivery as the experimental group’s experience. Otherwise, the experience should be considered “passive control.” | 0 = Passive control (i.e., typical academic/testing experience), 1 = Active control |
| Intervening, Moderator, and Mediator Variable Information | | | |
| Z | Level of self-identification with construct (as reported by authors) | Note: For studies/designs that have non-Black participants, this should be the level of self-identification with the construct among African Americans.  If authors do not report percentages of people who self-identify with the construct, then the proportion will be estimated “Some,” “Most,” or “All” by using the mean and standard deviation to estimate (under a normal distribution) the proportion of sample members who score at or above the midpoint of the self-identification scale. | Proportion of sample members who report self-identifying with construct. |
| AA | Task difficulty (as reported by authors) | Note: For studies/designs that have non-Black participants, this should be the difficulty of the task for African Americans.  If authors do not report exact percentages, but do label the task as “easy,” “medium difficulty,” or “hard,” (or similar terminology), then this will be coded. | 0 = Easy (mean score of 80% or higher) , 1 = Medium (mean score of 51-79%), 2 = Hard (mean score of 0-50%) |
| AB | Stereotype awareness (as reported by authors) | Note: For studies/designs that have non-Black participants, this should be the proportion of African Americans who are aware of the stereotype.  If awareness is reported via a rating scale, then “awareness” will be defined as scoring above the midpoint on the scale. Percentages of sample members above the midpoint may be estimated by using the mean and standard deviation (under a normal distribution). | Proportion of sample members who report being aware of the stereotype |
| AC | Stereotype belief (as reported by authors) | Note: For studies/designs that have non-Black participants, this should be the proportion of African Americans who believe the stereotype.  If belief/endorsement is reported via a rating scale, then “belief” will be defined as scoring above the midpoint on the scale. Percentages of sample members above the midpoint may be estimated by using the mean and standard deviation (under a normal distribution). | Proportion of sample members who endorse or state that they believe the stereotype to be true. |
| AD | Anxiety (as reported by authors) | Note: For studies/designs that have non-Black participants, this should be the proportion of African Americans who demonstrate anxiety.  If anxiety is reported via a rating scale, then “anxiety” will be defined as scoring above the midpoint on the scale. Percentages of sample members above the midpoint may be estimated by using the mean and standard deviation (under a normal distribution). | Proportion of sample members who exhibit or report having anxiety about the task. |
| AE | Average level of motivation for participants (as reported by authors) | Note: For studies/designs that have non-Black participants, this should be the average level of motivation in African Americans.  Scale will be divided into thirds. A mean or median in the lowest third of the motivation scale will be defined as “low motivation.” A mean or median in the middle third of the scale will be defined as “medium motivation.” A mean in the top third of the scale will be defined as “high motivation.” | 0 = Low, 1 = Medium, 2 = High |
| Dependent Variable Information | | | |
| AF | Name of measure of the dependent variable | DV should be a measure of academic or cognitive aptitude or achievement. |  |
| AG | Construct measured by the dependent variable |  | 0 = Specific academic achievement, 1 = General academic achievement, 2 = Specific academic aptitude, 3 = Intelligence or IQ, 4 = Other |
| AH | Authors’ name for the construct measured by the dependent variable |  |  |
| AI | Dependent variable type |  | 0 = Subjective rating, 1 = Experimenter-created test score, 2 = Single course grade, 3 = Multiple course grades (e.g., GPA), 4 = Standardized test altered by experimenter, 5 = Standardized test score for unaltered test, 6 = Other |
| AJ | Description of “Other” dependent variable type |  |  |
| AK | Test scorer status |  | 0 = Objective, 1 = Blind, 2 = Non-blind |
| AL | Stakes of dependent variable |  | 0 = No/low stakes, 1 = High stakes for course grade, 2 = Non-grade incentive for high performance |
| Sample Characteristics | | | |
| AM | Sample type |  | 0 = Community, 1 = College or university students, 2 = K-12 students, 3 = Other |
| AN | Description of “Other” sample type |  |  |
| AO | Median or mean sample socioeconomic status |  | 0 = Low, 1 = Medium or middle class, 2 = High |
| AP | Population demographics |  | 0 = African Americans are a minority of community or school, 1 = African Americans are a majority of community or school, 2 = Academic program designed for African American students, 3 = Other |
| AQ | Description of “other” population demographics |  |  |
| AR | Academic selectivity of sample |  | 0 = Academically struggling group, 1 = Academically typical/mixed/general group, 2 = Academically elite group |
| AS | Non-stereotyped group | Description of the non-stereotyped group members in the study. |  |
| AT | Total sample size | Not counting excluded participants. |  |
| AU | Sample size of Black intervention group | Not counting excluded participants. |  |
| AV | Sample size of Black control group | Not counting excluded participants. |  |
| AW | Sample size of non-stereotyped intervention group | Not counting excluded participants. |  |
| AX | Sample size of non-stereotyped control group | Not counting excluded participants. |  |
| AY | Total number of Black males in sample |  |  |
| AZ | Total number of Black females in sample |  |  |
| BA | Total number of non-stereotyped males in sample |  |  |
| BB | Total number of non-stereotyped females in sample |  |  |
| Results | | | |
| BC | Pre-test mean for Black intervention group |  |  |
| BD | Pre-test SD for Black intervention group |  |  |
| BE | Post-test mean for Black intervention group |  |  |
| BF | Post-test SD for Black intervention group |  |  |
| BG | Pre-test mean for Black control group |  |  |
| BH | Pre-test SD for Black control group |  |  |
| BI | Post-test mean for Black control group |  |  |
| BJ | Post-test SD for Black control group |  |  |
| BK | Pre-test mean for non-stereotyped intervention group |  |  |
| BL | Pre-test SD for non-stereotyped intervention group |  |  |
| BM | Post-test mean for non-stereotyped intervention group |  |  |
| BN | Post-test SD for non-stereotyped intervention group |  |  |
| BO | Pre-test mean for non-stereotyped control group |  |  |
| BP | Pre-test SD for non-stereotyped control group |  |  |
| BQ | Post-test mean for non-stereotyped control group |  |  |
| BR | Post-test SD for non-stereotyped control group |  |  |
| BS | Reported interaction effect size | Post-test dependent variable only, time x race interaction only |  |
| BT | Reported interaction effect size value | Post-test dependent variable only, time x race interaction only |  |
| BU | Reported interaction *p*-value | Post-test dependent variable only, time x race interaction only |  |
| BV | Reported interaction *p*-value > .05 interpreted to support stereotype threat theory? |  | 0 = No, 1 = Yes |
| BW | Recalculated interaction effect size | Post-test dependent variable only, time x race interaction only |  |
| BX | Recalculated interaction effect size value | Post-test dependent variable only, time x race interaction only |  |
| BY | Recalculated interaction *p*-value | Post-test dependent variable only, time x race interaction only |  |
| Statistical power | | | |
| BZ | Authors reported statistical power for the interaction? | Statistical power must be a priori power (not post hoc power), time x race interaction only | 0 = No, 1 = Yes |
| CA | Authors’ a priori effect size value used to calculate statistical power for the interaction | Time x race interaction only |  |
| CB | Authors’ reported a priori statistical power for the interaction | Time x race interaction only |  |
| CC | Recalculated a priori statistical power | Effect size for this calculation is η^2^ = .001 and a null main effect. |  |
| Data/Sample Member Exclusion | | | |
| CD | Were sample members excluded? |  | 0 = No, 1 = Yes |
| CE | Reason(s) for sample member exclusion |  |  |
| CF | Were DV(s) excluded? |  | 0 = No, 1 = Yes |
| CG | Names of DV(s) excluded |  |  |
| CH | Reason(s) that DV(s) were excluded |  |  |
| Covariate Control | | | |
| CI | Researcher controlled for covariates or confounding variables | This does *not* include using random assignment to control for confounding variables between intervention and control groups. | 0 = No, 1 = Yes |
| CJ | Primary method for controlling for covariates or confounding variables |  | 0 = ANCOVA, 1 = Matching, 2 = Regression, 3 = Other |
| CL | Description of “Other” primary method for controlling for covariates or confounding variables |  |  |
| CM | Multiple methods for controlling for covariates or confounding variables present? |  | 0 = No, 1 = Yes |
| CN | Names of other methods used to control for covariates or confounding variables | This does not include the primary method. |  |
| Miscellaneous Notes | | | |
| CO | Notes |  |  |

**Race x time x group interaction: Between-groups design (comparing groups of African Americans and non-stereotyped individuals)**

| Column | Label | Definition | Values |
| --- | --- | --- | --- |
| Document/Study Information | | | |
| A | Document No. |  |  |
| B | Study No. |  |  |
| C | Effect Size No. |  |  |
| D | Author(s) |  |  |
| E | Year |  |  |
| F | Author label for study | Appropriate if multiple studies are reported in the same article/manuscript. |  |
| G | DOI link or URL |  |  |
| H | Document Title |  |  |
| I | Type of Document |  | 0 = Published article, 1 = Pre-print, 2 = Dissertation/Thesis, 3 = Other Public Report, 4 = Uncirculated document/data |
| J | Sample description | Description of sample members |  |
| K | Pre-registered? | Describes whether the study was pre-registered before data collection | 0 = No, 1 = Yes |
| L | URL of pre-registration |  |  |
| M | Pre-registration recorded before data collection? |  | 0 = No, 1 = Yes |
| N | Pre-registration followed | Final study methodology must be close to the description in the pre-registration; any departures must be minor and/or explained and justified in the report. | 0 = No, 1 = Yes |
| O | Replication? | Author(s) described the study as a replication of a specific earlier stereotype threat study | 0 = No, 1 = Yes |
| P | Citation of study being replicated |  |  |
|  | Setting | | |
| Q | Setting |  | 0 = Laboratory or artificial setting, 1 = Subjects’ school or classroom, 2 = Clinic, 3 = Home, 4 = Other |
| R | Individual or group setting | Records whether subjects were exposed to the stereotype threat intervention individually or in a group setting | 0 = Individually, 1 = Group |
| S | Average proportion of subject group that was Black | Proportion of people in the group exposed to the stereotype intervention who were African American |  |
| Independent Variable Information | | | |
| T | Random assignment level |  | 0 = Individual, 1 = Group |
| U | Type of stereotype threat intervention | Stereotype threat triggers are designed to create the stereotype threat phenomenon in sample members. Mitigation interventions are designed to eliminate or reduce the impact of stereotype threat that is presumed to already be present. | 0 = Trigger, 1 = Mitigation, 2 = Other |
| V | Stereotype threat intervention description | Authors’ description of the stereotype threat intervention |  |
| W | Time lag between intervention and post-test | Recorded in days (if same day, report 0) |  |
| X | Manipulation check |  | 0 = None mentioned, 1 = Present and unsuccessful, 2 = Present and successful |
| Y | Control group experience | To be an “active control” experience, control group members must experience a placebo intervention of similar duration and delivery as the experimental group’s experience. Otherwise, the experience should be considered “passive control.” | 0 = Passive control (i.e., typical academic/testing experience), 1 = Active control |
| Intervening, Moderator, and Mediator Variable Information | | | |
| Z | Level of self-identification with construct (as reported by authors) | Note: For studies/designs that have non-Black participants, this should be the level of self-identification with the construct among African Americans.  If authors do not report percentages of people who self-identify with the construct, then the proportion will be estimated “Some,” “Most,” or “All” by using the mean and standard deviation to estimate (under a normal distribution) the proportion of sample members who score at or above the midpoint of the self-identification scale. | Proportion of sample members who report self-identifying with construct. |
| AA | Task difficulty (as reported by authors) | Note: For studies/designs that have non-Black participants, this should be the difficulty of the task for African Americans.  If authors do not report exact percentages, but do label the task as “easy,” “medium difficulty,” or “hard,” (or similar terminology), then this will be coded. | 0 = Easy (mean score of 80% or higher) , 1 = Medium (mean score of 51-79%), 2 = Hard (mean score of 0-50%) |
| AB | Stereotype awareness (as reported by authors) | Note: For studies/designs that have non-Black participants, this should be the proportion of African Americans who are aware of the stereotype.  If awareness is reported via a rating scale, then “awareness” will be defined as scoring above the midpoint on the scale. Percentages of sample members above the midpoint may be estimated by using the mean and standard deviation (under a normal distribution). | Proportion of sample members who report being aware of the stereotype |
| AC | Stereotype belief (as reported by authors) | Note: For studies/designs that have non-Black participants, this should be the proportion of African Americans who believe the stereotype.  If belief/endorsement is reported via a rating scale, then “belief” will be defined as scoring above the midpoint on the scale. Percentages of sample members above the midpoint may be estimated by using the mean and standard deviation (under a normal distribution). | Proportion of sample members who endorse or state that they believe the stereotype to be true. |
| AD | Anxiety (as reported by authors) | Note: For studies/designs that have non-Black participants, this should be the proportion of African Americans who demonstrate anxiety.  If anxiety is reported via a rating scale, then “anxiety” will be defined as scoring above the midpoint on the scale. Percentages of sample members above the midpoint may be estimated by using the mean and standard deviation (under a normal distribution). | Proportion of sample members who exhibit or report having anxiety about the task. |
| AE | Average level of motivation for participants (as reported by authors) | Note: For studies/designs that have non-Black participants, this should be the average level of motivation in African Americans.  Scale will be divided into thirds. A mean or median in the lowest third of the motivation scale will be defined as “low motivation.” A mean or median in the middle third of the scale will be defined as “medium motivation.” A mean in the top third of the scale will be defined as “high motivation.” | 0 = Low, 1 = Medium, 2 = High |
| Dependent Variable Information | | | |
| AF | Name of measure of the dependent variable | DV should be a measure of academic or cognitive aptitude or achievement. |  |
| AG | Construct measured by the dependent variable |  | 0 = Specific academic achievement, 1 = General academic achievement, 2 = Specific academic aptitude, 3 = Intelligence or IQ, 4 = Other |
| AH | Authors’ name for the construct measured by the dependent variable |  |  |
| AI | Dependent variable type |  | 0 = Subjective rating, 1 = Experimenter-created test score, 2 = Single course grade, 3 = Multiple course grades (e.g., GPA), 4 = Standardized test altered by experimenter, 5 = Standardized test score for unaltered test, 6 = Other |
| AJ | Description of “Other” dependent variable type |  |  |
| AK | Test scorer status |  | 0 = Objective, 1 = Blind, 2 = Non-blind |
| AL | Stakes of dependent variable |  | 0 = No/low stakes, 1 = High stakes for course grade, 2 = Non-grade incentive for high performance |
| Sample Characteristics | | | |
| AM | Sample type |  | 0 = Community, 1 = College or university students, 2 = K-12 students, 3 = Other |
| AN | Description of “Other” sample type |  |  |
| AO | Median or mean sample socioeconomic status |  | 0 = Low, 1 = Medium or middle class, 2 = High |
| AP | Population demographics |  | 0 = African Americans are a minority of community or school, 1 = African Americans are a majority of community or school, 2 = Academic program designed for African American students, 3 = Other |
| AQ | Description of “other” population demographics |  |  |
| AR | Academic selectivity of sample |  | 0 = Academically struggling group, 1 = Academically typical/mixed/general group, 2 = Academically elite group |
| AS | Non-stereotyped group | Description of the non-stereotyped group members in the study. |  |
| AT | Total sample size | Not counting excluded participants. |  |
| AU | Sample size of Black intervention group | Not counting excluded participants. |  |
| AV | Sample size of Black control group | Not counting excluded participants. |  |
| AW | Sample size of non-stereotyped intervention group | Not counting excluded participants. |  |
| AX | Sample size of non-stereotyped control group | Not counting excluded participants. |  |
| AY | Total number of Black males in sample |  |  |
| AZ | Total number of Black females in sample |  |  |
| BA | Total number of non-stereotyped males in sample |  |  |
| BB | Total number of non-stereotyped females in sample |  |  |
| Results | | | |
| BC | Pre-test mean for Black intervention group |  |  |
| BD | Pre-test SD for Black intervention group |  |  |
| BE | Post-test mean for Black intervention group |  |  |
| BF | Post-test SD for Black intervention group |  |  |
| BG | Pre-test mean for Black control group |  |  |
| BH | Pre-test SD for Black control group |  |  |
| BI | Post-test mean for Black control group |  |  |
| BJ | Post-test SD for Black control group |  |  |
| BK | Pre-test mean for non-stereotyped intervention group |  |  |
| BL | Pre-test SD for non-stereotyped intervention group |  |  |
| BM | Post-test mean for non-stereotyped intervention group |  |  |
| BN | Post-test SD for non-stereotyped intervention group |  |  |
| BO | Pre-test mean for non-stereotyped control group |  |  |
| BP | Pre-test SD for non-stereotyped control group |  |  |
| BQ | Post-test mean for non-stereotyped control group |  |  |
| BR | Post-test SD for non-stereotyped control group |  |  |
| BS | Reported interaction effect size | Post-test dependent variable only, race x time x group interaction only |  |
| BT | Reported interaction effect size value | Post-test dependent variable only, race x time x group interaction only |  |
| BU | Reported interaction *p*-value | Post-test dependent variable only, race x time x group interaction only |  |
| BV | Reported interaction *p*-value > .05 interpreted to support stereotype threat theory? |  | 0 = No, 1 = Yes |
| BW | Recalculated interaction effect size | Post-test dependent variable only, race x time x group interaction only |  |
| BX | Recalculated interaction effect size value | Post-test dependent variable only, race x time x group interaction only |  |
| BY | Recalculated interaction *p*-value | Post-test dependent variable only, race x time x group interaction only |  |
| Statistical power | | | |
| BZ | Authors reported statistical power for the interaction? | Statistical power must be a priori power (not post hoc power), race x time x group interaction only | 0 = No, 1 = Yes |
| CA | Authors’ a priori effect size value used to calculate statistical power for the interaction | Race x time x group interaction only |  |
| CB | Authors’ reported a priori statistical power for the interaction | Race x time x group interaction only |  |
| CC | Recalculated a priori statistical power | Effect size for this calculation is η^2^ = .001 and a null main effect and null two-way interactions. |  |
| Data/Sample Member Exclusion | | | |
| CD | Were sample members excluded? |  | 0 = No, 1 = Yes |
| CE | Reason(s) for sample member exclusion |  |  |
| CF | Were DV(s) excluded? |  | 0 = No, 1 = Yes |
| CG | Names of DV(s) excluded |  |  |
| CH | Reason(s) that DV(s) were excluded |  |  |
| Covariate Control | | | |
| CI | Researcher controlled for covariates or confounding variables | This does *not* include using random assignment to control for confounding variables between intervention and control groups. | 0 = No, 1 = Yes |
| CJ | Primary method for controlling for covariates or confounding variables |  | 0 = ANCOVA, 1 = Matching, 2 = Regression, 3 = Other |
| CK | Description of “Other” primary method for controlling for covariates or confounding variables |  |  |
| CL | Multiple methods for controlling for covariates or confounding variables present? |  | 0 = No, 1 = Yes |
| CM | Names of other methods used to control for covariates or confounding variables | This does not include the primary method. |  |
| Miscellaneous Notes | | | |
| CN | Notes |  |  |
