# Supplementary material for: Protocol for a meta-analysis of stereotype threat in African Americans
Source: PLoS One. 2024 Jul 24;19(7):e0306030. doi: 10.1371/journal.pone.0306030 (PMC11268653; doi:10.1371/journal.pone.0306030)
Supplement: S2 Appendix — (DOCX) [file pone.0306030.s003.docx]

**Appendix B**

**Variable Section Type of Moderator Reason for Inclusion Reference(s)**

| Variable | Section of Coding Document | Type of Moderator | Explanation for Inclusion | Selected Relevant Reference(s) |
| --- | --- | --- | --- | --- |
| Type of Document | Document/Study Information | Methodological | Publication bias has been detected in past meta-analyses of stereotype threat research, which means journal articles may have inflated effect sizes compared to than unpublished studies | Flore & Wicherts (2015); Ganley et al. (2013); Shewach et al. (2019); Warne (2022); Zigerell (2017) |
| Pre-registered? | Document/Study Information | Methodological | Pre-registration is designed to reduce questionable research practices that inflate Type I error rates | Moore (2016) |
| Replication? | Document/Study Information | Methodological | Replications often have smaller effect sizes than original studies | Open Science Collaboration (2015) |
| Setting | Setting | Methodological | Ecologically valid studies tend to show smaller effect sizes for stereotype threat | Shewach et al. (2019) |
| Individual or Group Setting | Setting | Methodological | Group settings have been theorized to be more likely to trigger stereotype threat (e.g., if non-stereotyped group members are present, or if a person is a minority in a group) | Flore & Wicherts (2015) |
| Random assignment level | Independent Variable Information | Methodological | Random assignment at the group level will create clustering of observations, which may lead to erroneous standard errors (and inflated Type I error) if intraclass correlations are > 0. | Warne et al. (2012) |
| Type of stereotype threat intervention | Independent Variable Information | Theoretical | Triggers and mitigations are different experimental interventions given for different reasons. This introduces heterogeneity into the stereotype threat literature | Spencer et al. (2016) |
| Manipulation check | Independent Variable Information | Methodological | Manipulation checks are often an important part of ensuring that an intervention was effective | Kane & Barabas (2019) |
| Control group experience | Independent Variable Information | Methodological | An active control group experience can reduce expectancy effects and make groups more comparable | Boot et al. (2013) |
| Level of self-identification with construct | Intervening, Moderator, and Mediator Variable Information | Theoretical | Theorized to be one of the “essential” conditions for stereotype threat to occur | Steele (1997) |
| Task difficulty | Intervening, Moderator, and Mediator Variable Information | Theoretical | Theorized to be one of the “essential” conditions for stereotype threat to occur | Steele (1997) |
| Stereotype awareness | Intervening, Moderator, and Mediator Variable Information | Theoretical | Theorized to be one of the “essential” conditions for stereotype threat to occur | Steele (1997) |
| Stereotype belief | Intervening, Moderator, and Mediator Variable Information | Theoretical | Belief in a stereotype is not theorized to be important for triggering stereotype threat | Steele (1997) |
| Anxiety | Intervening, Moderator, and Mediator Variable Information | Theoretical | Stereotype threat has been theorized to operate by inducing anxiety in subjects | Hollis-Sawyer & Sawyer (2008); Pennington et al. (2016) |
| Average level of motivation for participants | Intervening, Moderator, and Mediator Variable Information | Theoretical | Motivation has been theorized to function as a mitigating variable for stereotype threat | Pennington et al. (2016) |
| Construct measured by the main dependent variable | Dependent Variable Information | Theoretical | Measures of achievement and aptitude vary in their susceptibility to change. | Starr & Riemann (2022) |
| Dependent variable type | Dependent Variable Information | Theoretical, Empirical | Measures of academic and cognitive performance vary in the strength of their relationship to non-cognitive variables. Also, stereotype threat effect sizes may be stronger for standardized test scores than unstandardized dependent variables. | Lecher et al. (2017); Picho-Kiroga et al. (2021) |
| Test scorer status | Dependent Variable Information | Methodological | Scores from blinded raters are likely less biased than those from unblinded raters. | Marcus et al. (2005) |
| Stakes of dependent variable | Dependent Variable Information | Empirical | Ecologically valid studies tend to show smaller effect sizes for stereotype threat | Shewach et al. (2019) |
| Sample type | Sample Characteristics | Empirical | Strength of the stereotype threat phenomenon may vary across age groups | Picho-Kiroga et al. (2021) |
| Median or mean sample socioeconomic status | Sample Characteristics | Exploratory | We are interested in whether socioeconomic status could be a moderator variable in stereotype threat research. | — |
| Population demographics | Sample Characteristics | Theoretical | Minority salience may trigger threats in people | Murphy et al. (2007) |
| Academic selectivity of sample | Sample Characteristics | Theoretical | High cognitive ability and high prior academic performance are strong predictors of high performance on academic and cognitive dependent variables. This may make academic selectivity a confounding or covariate variable for stereotype threat studies. | Ackerman et al. (2013); Zaboski et al. (2018) |
| Were sample members excluded? | Data/Sample Member Exclusion | Methodological | Sample member exclusion can be a questionable research practice | John et al. (2012) |
| Were DVs excluded? | Data/Sample Member Exclusion | Methodological | Dependent variable exclusion can be a questionable research practice | John et al. (2012), Simmons et al. (2011) |
| Researcher controlled for covariates or confounding variables | Covariate Control | Methodological | Controlling for covariates or confounding variables can be a questionable research practice | Simmons et al. (2011) |
| Primary method for controlling for covariates or confounding variables | Covariate Control | Exploratory | We are interested in whether any covariate control has a systematic influence on effect sizes. | — |
| Multiple methods for controlling for covariates or confounding variables present? | Covariate Control | Exploratory | We are interested in whether using multiple covariate control methods has a systematic influence on effect sizes. | — |

Note: The references in the far-right column are not intended to be comprehensive nor to indicate the earliest discussion of a moderator in the stereotype threat literature.
