# Supplementary material for: Protocol for a meta-analysis of stereotype threat in African Americans
Source: PLoS One. 2024 Jul 24;19(7):e0306030. doi: 10.1371/journal.pone.0306030 (PMC11268653; doi:10.1371/journal.pone.0306030)
Supplement: S3 Appendix — (DOCX) [file pone.0306030.s004.docx]

**Appendix C**

**Multilevel Framework for Analysis**

In our meta-analysis, a multilevel model is employed to account for the potential existence of multiple effect sizes within each study, indicating that effect sizes within a study are not independent. This approach enables the estimation of both within-study and between-study variations in effect sizes. Note, these equations only assume two levels but the logic can easily be extended to three levels.

In the simplest case where a study includes only one racial group and one treatment and trigger the simple linear regression equation would look thusly:

*y_i_=𝛽_0_  + 𝛽_1_(Intervention)_i_ + ε_i_*

we denote an observed effect size for individual study *i* as *y_i_*. The term *𝛽_0_* represents the average test scores from the control group, while *𝛽_1_*  signifies the treatment effect of an intervention aimed at mitigating stereotype threat or triggering stereotype threat. The residual for individual study effect size *i*, denoted as *ε_i_*, is derived from the difference between the expected and observed test scores for that individual study observation. To assess stereotype threat, *𝛽_0_* serves as a key parameter. It enables the calculation of the average test scores for an untreated group of African Americans, allowing for a meaningful comparison with a national average or other relevant statistical benchmarks.

But this model assumes that each data point is independent. It is probable that there are more than one effect sizes of stereotype threat included in the same study. These multiple estimates of stereotype threat would not be independent and thus would violate an assumption of the simple linear regression model. To account for this dependency in the data a multilevel simple linear regression model would be run with this equation:

*y_ij_=𝛽_0 jk_ + 𝛽_1_(Intervention)_ijk_ + ε_ijk_*

where *𝛽_0 jk_=𝛽_0k_ + r_jk_*

Where *i* = an individual effect size estimate, and *j* = individual study, and *k* = publication (e.g., journal dissertation, thesis, unpublished report, etc.). Thus, each study would have its own “random” intercept of stereotype threat for African-Americans (*𝛽_0 jk_)* and there would be an overall intercept of stereotype threat across all studies (*𝛽_0 k_*)*. r_jk_* is the deviance of each individual intercept from the overall intercept of all studies across all publications.

where *𝛽_0 k_=𝛽_00_ + u_k_*

where *𝛽_00_* is the overall stereotype threat across all publications and *u_k_* is the the deviance of each individual publication from the overall intercept.

In more complex scenarios, where the study encompasses multiple ethnic groups, the multilevel model can be expanded as follows:

*y_i_=𝛽_0_  + 𝛽_1_(Treatment or Trigger)_i_ + 𝛽_2_(African-American)_i_ + 𝛽_3_(African-American*Intervention)_i_ + ε_i_*

Where *𝛽_2_* specifically captures the effect of being African-American and serves as an estimate of the stereotype threat. *𝛽_3_* calculates the differential effect of the treatment or trigger on African-Americans. As in the simple linear regression model there may be and probably will be multiple estimates of stereotype threat for African-Americans within the same study violating the independence of observation assumption. As our parameter of interest is now a coefficient (*𝛽_2_)* that is not the intercept we need to run a random coefficient (or random slope) multilevel model which is given as:

*y_ijk_=𝛽_0jk_  + 𝛽_1jk_(Treatment or Trigger)_ijk_ + 𝛽_2jk_(African-American)_ijk_ + 𝛽_3j_(African-American*Intervention)_ijk_ + ε_ijk_*

*𝛽_2jk_= 𝛽_2k_* + *r_2jk_*

*𝛽_2k =_ 𝛽_200_ +u_2k_*

Now *𝛽_2jk_* is the estimate of stereotype threat for African-American for study *j* publication *k* and *𝛽_2k_* is the the overall estimate stereotype threat across all studies within a publication of African-Americans across all studies. *r_2jk_* is the deviance of each study’s stereotype estimate with the overall average within a publication. *𝛽_2k_* and  *u_2k_* are the intercepts and deviance terms for at the publication level. The parameter of interest in the meta-analysis is *𝛽_200_*, the estimate of stereotype threat for African-Americans across all studies.
